# Bioderived Cellulose Aerogel Fibers with Hierarchical Porosity for Millisecond Hydrovoltaic Sensing

**DOI:** 10.34133/research.1324

**Published:** 2026-06-09

**Authors:** Lanyue Zhang, Yuqing Xie, Shiang Shi, Xiang Chen, Xiaotong Fu, Qichun Feng, Dongdong Ye

**Affiliations:** School of Materials and Chemistry, Anhui Agricultural University, Hefei, Anhui Province 230036, China.

## Abstract

Real-time moisture sensing is crucial for environmental monitoring and food safety. However, conventional humidity sensors relying on moisture-induced changes in conductivity or capacitance are often constrained by sluggish water adsorption–desorption kinetics and inefficient ion transport through disordered nanochannels. Here, we develop cellulose aerogel fibers derived from natural rattan, featuring hierarchical and interconnected porosity, to enable ultrafast hydrovoltaic moisture sensing. The tailored pore network expedites the diffusion of trace moisture and substantially amplifies the solid–liquid interfacial area within the fiber, thereby boosting the formation of electric double layers and enhancing water-ion coupling. Consequently, the self-powered sensor delivers a detectable voltage response within 70 ms upon exposure to only 10 μl of water microdroplets, achieving both rapid detection and high sensitivity. Moreover, the device exhibits versatile responsiveness to multiple environmental parameters, and its sensing behavior can be readily tuned by manipulating the fiber configuration (linear, helical, and branched). All 3 configurations achieve a fast response to approximately 0.1 V within 70 to 310 ms, followed by a voltage increase to 0.49 to 0.63 V within 3 to 5 s. This work highlights the critical role of porosity engineering in cellulose aerogel fibers and provides a new strategy for developing high-performance biomass-based hydrovoltaic sensing systems.

## Introduction

Moisture sensing plays a crucial role in environmental monitoring, material protection, and the preservation of sensitive commodities [1–4]. In particular, grain serves as a fundamental source of nutrients, and its stability during storage and transportation depends heavily on moisture control, as excess moisture can trigger deterioration processes such as mold growth, biochemical degradation, and structural instability [5–7]. Accurate and timely moisture detection is therefore critical for preventing quality loss and ensuring safe handling [8]. However, most existing humidity sensors rely on moisture-induced changes in electrical conductivity or capacitance, mechanisms that fundamentally depend on water adsorption–desorption cycles and ion migration within the sensing medium [9–11]. These processes are intrinsically slow and often constrained by disordered nanochannels, resulting in delayed responses, insufficient sensitivity to trace moisture, and difficulties in scaling devices toward miniaturized, rapid-response formats[12–14].

Hydrovoltaic technology offers a promising pathway for self-powered moisture detection by converting moisture-driven ion movement into electrical signals [15–17]. Among these systems, water-evaporation-induced electricity generators are particularly representative [18–20]. Their power generation relies on capillary-driven water transport [21], continuous evaporation within nanochannels [22,23], and the directional migration of oppositely charged ions under the influence of the electric double layer (EDL) [24] establishing an ion gradient that produces a measurable potential and current [25]. However, in conventional materials such as ordinary cellulose paper, natural cellulose films, and unmodified polyelectrolyte hydrogels, the ion–water diffusion equilibrium typically requires minutes to reach equilibrium due to high flow resistance, gravity-induced effects, and disordered structures [26,27]. This severely restricts sensor response speed and reduces detection accuracy under low-moisture conditions (ambient relative humidity ≤ 20%). These challenges highlight the need for rationally designed nanochannel architectures that accelerate moisture transport, enhance charge separation, and strengthen water–ion coupling, thereby enabling ultrafast and high-efficiency hydrovoltaic sensing [28–32].

Inspired by efficient transport structures in nature, we design porosity-engineered cellulose aerogel fibers derived from natural rattan to achieve ultrafast hydrovoltaic moisture sensing. Through controlled regeneration, the fibers develop continuously aligned, hierarchically interconnected pores that enable rapid moisture diffusion and directional ion migration, substantially increasing the solid–liquid interfacial area and accelerating the formation of EDLs. As a result, only 10 μl of test liquid generates an open-circuit voltage of 0.05 V within 70 ms, which stabilizes at 0.61 V within 1.9 s. Beyond rapid response and high sensitivity, the fibers exhibit multisensory capabilities in response to humidity, airflow, temperature, and light. Tailored structural configurations further enable tunable sensing behaviors, enabling flexible optimization between the monitoring range and response speed. This work demonstrates the effectiveness of porosity engineering in cellulose aerogel fibers and offers a promising strategy for self-powered, high-performance hydrovoltaic moisture sensors.

## Results

### Bioinspired construction of porosity-engineered cellulose aerogel fibers

**Fig. 1.**
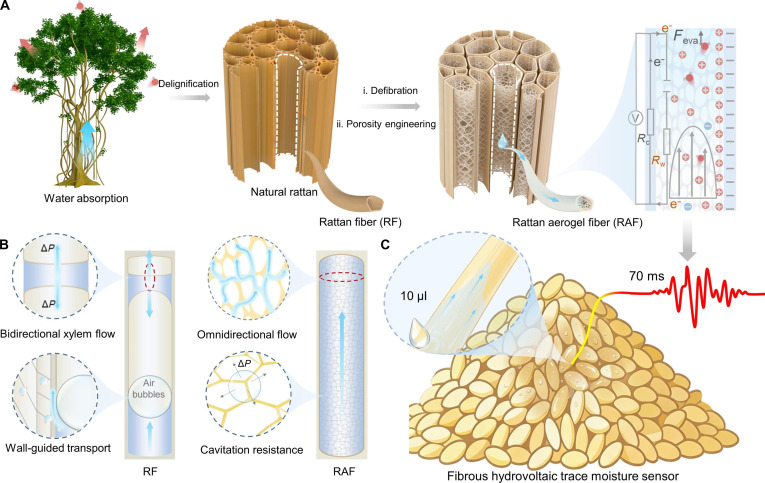
Water transport pathways and structural organization in rattan fiber (RF) and rattan aerogel fiber (RAF). (A) Process flow diagram for RAF fabrication and the mechanism of streaming potential formation. (B) Water transport behavior of RF and RAF. (C) Schematic diagram of the operation of a fibrous hydrovoltaic trace moisture sensor.

Inspired by the hierarchical transport architecture of natural rattan, we developed a regeneration process to reconstruct an individual rattan fiber (RF) into a regenerated rattan aerogel fiber (RAF) featuring a nanoscale, 3-dimensional (3D) interconnected network within the lumen (Fig. 1A). By selectively removing lignin and hemicellulose from the rattan cell walls (Fig. S1), the material is defibrated into negatively charged micrometer-scale xylem vessels and nanoscale cellulose microfibrils (Figs. S2 to S4), from which RF is isolated. Subsequent partial dissolution leads to the diffusion of dissolved cell wall components into the lumen, followed by aqueous precipitation, forming a nanoscale network that is intimately integrated with the vessel wall (Fig. 1A). This reconstructed porous framework introduces numerous parallel pathways and significantly enhances fluid accessibility within the cavity.

The different internal structures of RF and RAF result in distinct water-transport behaviors. When RF is used for trace water transport, the water column is highly susceptible to disruption by bubble formation, leading to intermittent flow and severely compromised transport efficiency [33,34]. Under these conditions, water is forced to bypass the blocked lumen and travels along the vessel wall. Upon reentering the lumen, the flow generates localized countercurrents in the direction opposite to the primary flow (Fig. 1B and Movie S1). As a result, the RF water transmission speed is only 0.047 mm/s (Fig. S5). In contrast, RAF effectively avoids bubble-induced discontinuity and supports rapid, directional, and stable water delivery at the single-fiber level (Fig. 1B and Movie S2). The internal multipath transmission architecture boosts water transport speed to 2.7 mm/s (Fig. S5), achieving nearly a 2-order-of-magnitude increase over RF. Due to its unidirectional, rapid water transport properties, RAF exhibits favorable functional responsiveness for energy harvesting. As water flows through its internal porous channels and undergoes phase change at the evaporation interface, the solid–liquid interaction between water molecules and the channel walls induces the formation of an EDL (Fig. 1A). This is because, after nanoengineering modification, the RAF surface is rich in carboxyl and phenolic hydroxyl groups, which dissociate in water to render the fiber surface negatively charged, forming a stable EDL. Driven synergistically by water evaporation and capillary force, water flows directionally along the hierarchically interconnected channels inside the fiber, thereby dragging the mobile cations in the EDL to migrate, which generates a streaming current. Under dynamic equilibrium, a stable streaming potential forms, converting the fluid motion induced by water evaporation into stable electrical signals, thereby enabling hydrovoltaic moisture sensing. Leveraging this mechanism, RAF can precisely detect trace amounts of moisture on grain surfaces and rapidly convert them into recognizable electrical signals, enabling rapid response detection of trace moisture (Fig. 1C).

### Water permeation and ultrafast transport characteristics of RAF

**Fig. 2.**
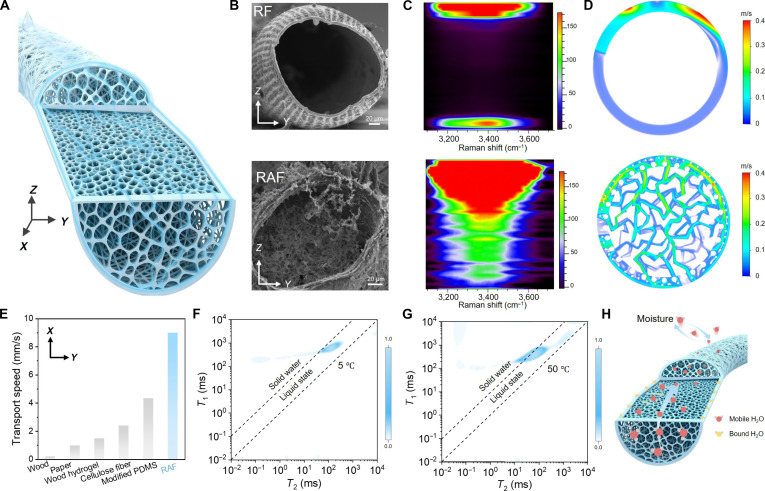
Enhanced water transport behavior and structural characterization of rattan aerogel fiber (RAF). (A) Schematic illustration of water-transport pathways within RAF. (B) Cross-sectional morphologies of RAF and rattan fiber (RF). (C) Two-dimensional (2D) Raman mapping images of RAF and RF. (D) Ansys Fluent simulations of water transport, showing the time evolution of flow velocity magnitude in RF and RAF. (E) Comparison of water-transport velocities across different substrates. PDMS, polydimethylsiloxane. (F and G) Low-field ^1^H *T*_1_−*T*_2_ revealing water dynamics in RAF at 5 and 50 °C. (H) Schematic representation of moisture exchange within RAF and its interaction with the surrounding environment.

The hierarchical porous architecture endows the RAF with exceptional permeability and water transport capability (Fig. 2A and Fig. S6). Scanning electron microscope characterization confirms that the natural RF maintains a hollow tubular lumen with a diameter of approximately 184 μm (Fig. 2B). In the RAF, the partial dissolution and regeneration process triggers the formation of a uniform cellulose nanofiber network within the lumen, resulting in a structurally homogeneous cross-section (Fig. 2B). According to the Barrett–Joyner–Halenda model calculation, the pore size of RAF is mainly concentrated in the typical mesoporous range of 2 to 50 nm, with a peak pore size of approximately 3 to 4 nm (Fig. S7), and its specific surface area is 119.46 m^2^/g (Fig. S8). To visualize the spatial distribution of trace moisture, we performed the 2D Raman imaging at 3,400 cm^−1^ [35]. The RF exhibits water-associated signals confined solely to its upper and lower surfaces, with the lumen showing nearly no detectable water signal (Fig. 2C). By contrast, the RAF exhibits uniform water distribution across the entire cross-section (Fig. 2C), indicating markedly improved moisture accessibility throughout the fiber interior.

ANSYS Fluent simulations further elucidate the transport behavior within the distinct fiber architectures (Fig. S9). In RF, water is predominantly confined to a narrow interface region near the inner wall, and the flow velocity decreases progressively along the transport pathway (Fig. 2D). Conversely, the interconnected nanofibrous network in RAF provides abundant multiscale permeation pathways. The resulting distributed pore channels maintain balanced flow velocities. At the same time, the dramatically increased solid–liquid interfacial area enhances capillary driving forces, thereby promoting moisture penetration along low-resistance routes (Fig. 2D). These simulations verify that the nanofiber network in RAF establishes efficient cross-sectional moisture transport pathways. Following the removal of lignin and hemicellulose, RAFs exhibit instantaneous moisture wetting, while the internal capillary forces generated by the cellulose nanofiber network enable continuous water pumping (Fig. S10). As a result, the moisture transport velocity of RAF reaches 9 mm/s (Fig. 2E), far exceeding the transport capability of common biomass materials, polymeric substrates, and natural wood (Table S1) [36–39].

Low-temperature nuclear magnetic resonance (NMR) characterizations reveal dynamic liquid–solid exchange behavior of water confined at semidisordered cellulose surfaces. When the *T*_1_/*T*_2_ ratio approaches 10, water exhibits properties intermediate between those of a liquid and a solid (Fig. 2F and Fig. S11) [40]. In addition, low-field NMR-derived water-orientation plots show a spindle-shaped distribution nearly aligned with the fiber diagonal (Fig. S12) [41]. Upon heating to 50 °C, the exchange between liquid and vapor phases intensifies, accelerating moisture release from the fiber interior (Fig. 2G). This temperature-dependent transition between liquid–solid and liquid–gas exchange modes enables RAF to sustain ion transport and continuous water, while simultaneously pumping under varying environmental conditions (Fig. 2H).

### Hydrovoltaic energy generation enhanced by porosity engineering

RAFs exhibit significant hydrovoltaic output driven by evaporation-induced electrokinetic processes. As water permeates the negatively charged, regenerated porous network, counterions accumulate at the solid–liquid interface, forming an EDL that generates a continuous streaming potential and current [42–44]. This mechanism was confirmed by mounting the fibers between a horizontal support and an electrolyte reservoir (Fig. S13). Reversing the connected electrodes resulted in a corresponding polarity reversal in the output voltage, while maintaining the same amplitude (Fig. S14), thereby verifying its intrinsic hydrovoltaic origin. Compared with RF, RAF delivers a significantly higher open-circuit voltage of approximately 0.61 V (Fig. S15). With increasing load resistance from 100 Ω to 1 MΩ, the voltage rises from 22 to 550 mV, while the current decreases from 0.13 to 0.00315 μA, reaching a maximum power density of 1.94 μW/cm^2^ (Fig. S16).

**Fig. 3.**
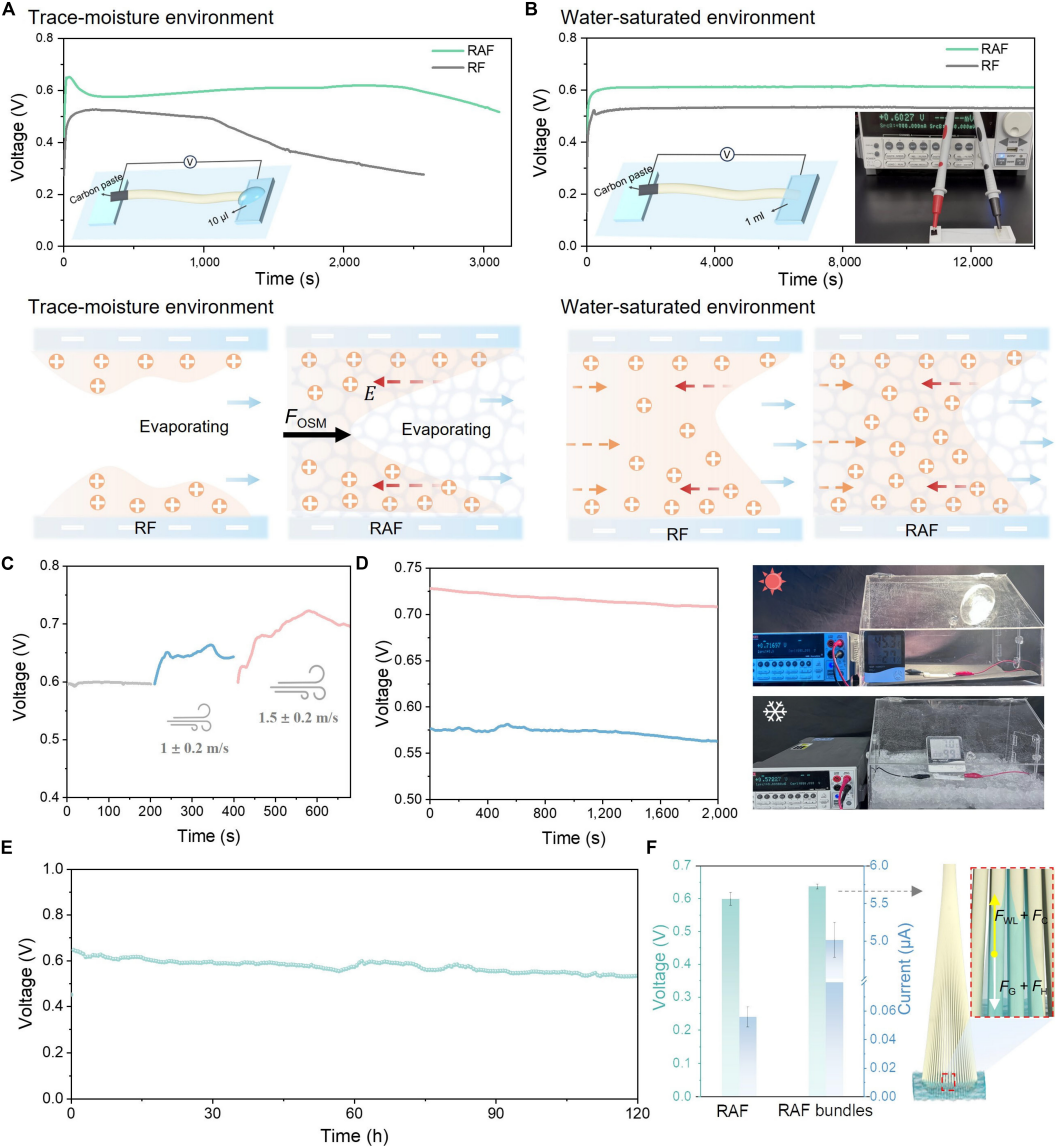
Electrical output behavior and nanochannel mechanisms of rattan aerogel fiber (RAF). (A) Voltage outputs of RAF and rattan fiber (RF) under trace-moisture (10 μl of 2.5 wt% NaCl solution) and water-saturated environments (1 ml of 2.5 wt% NaCl solution). (B) Schematic comparison of nanochannel configurations in RAF and RF under trace-moisture and water-saturated environments. (C) Voltage response of RAF under different airflow velocities. (D) Voltage output of the RAF at various ambient temperatures. (E) Open-circuit voltage of RAF recorded continuously for 120 h at ambient temperature (30 ± 2 °C; relative humidity, 27 ± 2%). (F) Output performance of RAF and an RAF bundle with different water-transport behavior. *F*_WL_, Laplace force; *F*_C_, capillary force; *F*_G_, gravitational force; *F*_H_, viscous force.

Under trace moisture input (10 μl of 2.5 wt% NaCl solution), RAF rapidly rises to 0.17 V within 0.17 s and reaches stabilization above 0.6 V within 1.8 s (Fig. S17). Its output persists for 3,127 s, which is significantly longer than RF (2,578 s) (Fig. 3A). This rapid onset and prolonged duration arise from the synergy of strong osmotic pressure and enhanced capillarity within the porous RAF channels, which efficiently transport ions toward the channel apex and generate a dense cation layer. The resulting electric field is further strengthened by shear flow within the nanofiber network. In contrast, RF lacks sufficient osmotic driving force (*F*_OSM_) to sustain moisture delivery to the electrode region, leading to premature signal decay (Fig. 3A). When the moisture supply increases (1 ml of NaCl solution), RAF and RF stabilize at 0.61 and 0.53 V, respectively, and maintain output for over 12,000 s (Fig. 3B). A dynamic equilibrium between evaporative flow, capillary-assisted water transport, and ion-induced Coulombic repulsion governs this long-term stability. Structurally, RF exhibits a flow pattern dominated by the central region, which limits proton enrichment near the walls, whereas RAF’s interconnected porous network dramatically increases the solid–liquid interfacial area and enhances EDL formation, thereby generating a stronger electric field (Fig. 3B). To further investigate the influence of the regenerated cellulose nanonetwork distribution on hydrovoltaic performance, we prepared samples with NaOH soaking times of 0, 3, 6, 12, and 24 h, which were designated as RAF-0, RAF-3, RAF-6, RAF-12, and RAF, respectively. The experimental results show that the output voltage gradually increases with the degree of regeneration (Fig. S18), indicating that the evaporation-driven hydrovoltaic output exhibits a significant structure-dependent enhancement.

Environmental conditions also affect the hydrovoltaic performance of RAF. At 50% relative humidity, the open-circuit voltage increases from 0.60 V under still air to 0.66 V at an airflow velocity of 1 m/s and further to 0.72 V at 1.5 m/s (Fig. 3C). RAF operates stably over a broad temperature range of 7 to 45 °C (Fig. 3D), and it delivers a continuous output for at least 5 d under uninterrupted operation (Fig. 3E). In contrast, elevated humidity suppresses evaporation and decreases the output voltage (Fig. S19). Owing to the coupled effects of capillary action and Laplace pressure in bifurcated microchannels, the RAF bundle produces a higher open-circuit voltage than a single fiber (Fig. 3F, Fig. S20, and Movie S3). The enlarged cross-sectional area also increases the current output. Furthermore, RAF units can be modularly assembled to scale both voltage and current. Specifically, 17 fibers connected in series generate ~10 V (Fig. S21), and capacitors ranging from 100 to 2,200 μF can be charged within 500 s (Fig. S22).

### Ultrafast and modular hydrovoltaic sensing systems

**Fig. 4.**
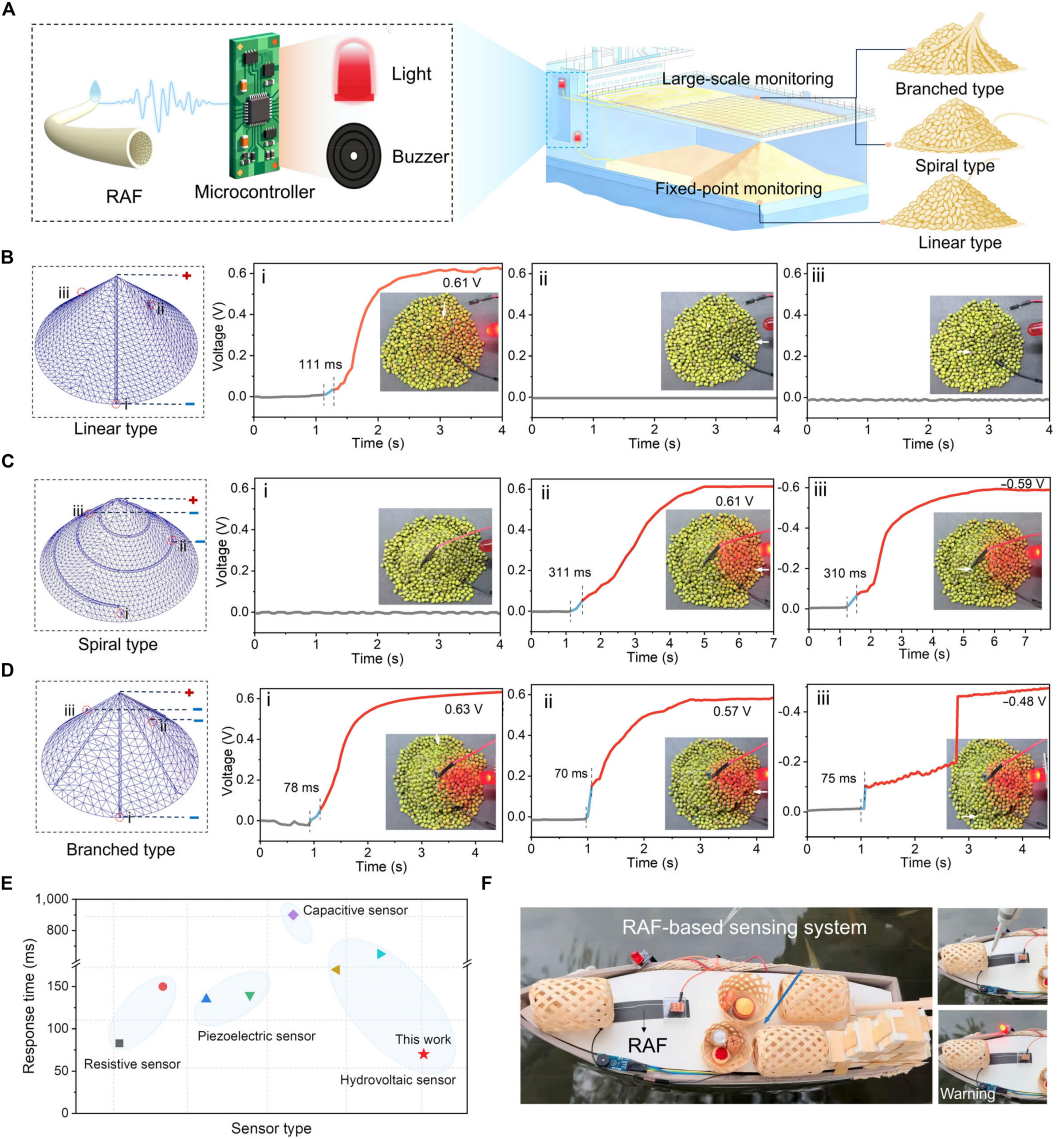
Trace-moisture sensing performance and application of rattan aerogel fiber (RAF). (A) Schematic illustration of the trace-moisture sensing system. (B) Voltage signals at points i, ii, and iii under linear configuration. (C) Voltage signals at points i, ii, and iii under spiral configuration. (D) Voltage signals at points i, ii, and iii under branched configuration. (E) Comparison of response times among representative moisture sensing systems. (F) Photograph of the RAF-based sensing device triggering an alarm during trace-moisture detection.

Trace surface moisture is a critical indicator of quality changes in stored or transported food commodities, especially during maritime logistics. Leveraging the high moisture sensitivity and rapid ionic response of RAF, we developed a modular trace-moisture monitoring system in which RAF serves as the sensing unit, while the moisture-induced electrical signal is processed in real time by a microcontroller to trigger an alarm integrating visual and acoustic outputs (Fig. 4A).

To accommodate varying monitoring conditions, we designed and constructed 3 structural configurations—linear, helical, and branched (Fig. 4A). The specific dimensions of the 3 sensor configurations are as follows: The linear sensor consists of a single fiber 4 cm in length; the helical sensor is made of a 16-cm fiber wound into a helix; and the branched sensor comprises 7 fibers, each 4 cm long, gathered at the top and dispersed at the bottom. All sensors were placed in a conical grain bin with a diameter of 5.5 cm and a height of 3.2 cm, and all 3 sensor types operated in 3D space. Using the center of the bin bottom as the origin (0, 0, 0), the 3 monitoring points were set at distances of 4, 2, and 1 cm from the top of the sensor fiber, with 3D coordinates as follows: point i (0, 2.95, 0.2), point ii (1.25, −0.9, 1.5), and point iii (−2.1, 0, 0.9). These 3 test points were selected to systematically meet the humidity detection requirements in different regions of the grain bin.

Comparative tests performed at 3 designated monitoring points (Fig. 4B to D) reveal that sensor geometry profoundly influences both coverage and response. At monitoring points ii and iii, both the branched and helical configurations responded effectively. Since opposite water-transport directions produced negative voltages, the absolute values were used for comparison. Although the maximum voltage change is around 0.6 V, it is sufficient for practical early-warning applications. Given the extremely low noise floor (<5 mV), the 0.1V alarm threshold provides excellent signal discrimination between dry and moisture-present states during grain storage. Owing to its optimized water-transport pathways, the branched sensor achieved ultrafast response times of 70 and 75 ms, substantially outperforming the helical counterpart, which required 311 and 310 ms, respectively. In contrast, the linear sensor remained unresponsive at these positions because of its limited coverage (Figs. S23 to S31). These results indicate that the linear configuration is suitable for localized detection, the helical design is more appropriate for large-area monitoring, and the branched architecture provides the best overall balance among sensing range, transport efficiency, and response speed.

The response time of the RAF hydrovoltaic sensing system was further benchmarked against representative moisture-sensing platforms based on resistive [45,46], piezoelectric [47,48], capacitive [49], and hydroelectric [13,29] principles. As summarized in Fig. 4E and Tables S2 and S3, the RAF-based sensor exhibited a rapid response time of 70 ms, outperforming most previously reported devices. Durability assessments show that RAF sensors maintain stable voltage output after repeated sensing–drying cycles at room temperature, indicating good reversibility and reusability rather than single-use operation (Fig. S32). Field-scale validation under real maritime shipping conditions further confirmed the robustness and applicability of the system, enabling successful detection of trace moisture in grain cargo and timely warning output (Fig. 4F and Movie S4). Overall, the RAF-based sensing system provides a sensitive, rapid, and reliable strategy for trace-moisture detection in maritime food logistics (Fig. S33), although it should be noted that the current design is not suitable for fully sealed, nonventilated environments or under extremely low-humidity conditions (<10% relative humidity).

## Discussion

The results collectively show that the key to the superior hydrovoltaic sensing performance of RAF lies in the reconstruction of the original hollow lumen into a hierarchically interconnected porous transport network. Compared with the single-cavity structure of RF, this regenerated architecture provides multiple parallel pathways for water penetration, greatly improves internal wettability, and suppresses bubble-induced transport interruption. These structural changes fundamentally alter the way in which trace moisture enters, redistributes, and migrates through the fiber, thereby transforming a poorly accessible lumen into a highly permeable ionic transport medium.

This architectural reconstruction also strengthens the coupling between water transport and charge generation. In RF, moisture is largely confined to the vessel wall or external surface, limiting effective ion accumulation at the solid–liquid interface and leading to unstable or delayed electrical output. In contrast, the nanofiber network in RAF increases the effective solid–liquid interface area across the entire cross-section due to its higher specific surface area (119.46 m^2^/g), facilitating rapid EDL formation upon water introduction. The more homogeneous moisture distribution observed by Raman mapping, together with the more balanced flow field predicted by simulation, indicates that pore engineering does not simply accelerate liquid uptake but also creates a more favorable environment for directional ion migration and streaming potential formation.

The low-field NMR results further suggest that confined water in RAF exists in a dynamic state between free and strongly bound water. Such semibound water likely reduces interfacial transport resistance while maintaining sufficient interaction with the charged cellulose surface, thereby supporting efficient ion motion under trace-moisture conditions [36]. At elevated temperature, the accelerated liquid–vapor exchange facilitates moisture release and renews the internal transport process. This dynamic interfacial water behavior helps explain why RAF maintains stable hydrovoltaic output across different temperatures and environmental conditions [37].

The marked improvement in hydrovoltaic output with increasing volume fraction of the regenerated cellulose nanonetwork within the pore structure further confirms that pore structure is a dominant design parameter. A higher volume fraction allows moisture to be redistributed more rapidly across the fiber interior, reduces hydraulic resistance, and increases the number of effective transport channels participating in electrokinetic conversion. As a result, the ion gradient established under evaporation can be sustained more efficiently, leading to a higher voltage output (0.61 V). The stronger response under airflow and the lower output at high humidity are also consistent with this mechanism, as the evaporation rate directly modulates the driving force for directional ion transport.

Importantly, the sensing results show that the porous RAF architecture is not only beneficial for power generation under continuous water supply but is especially effective under trace-moisture input. The ability to produce a detectable signal within 70 ms under minimal water exposure demonstrates that the system operates in a regime where moisture capture, interfacial activation, and ion migration occur on a highly compressed timescale. This is a major distinction from conventional resistive or capacitive moisture sensors (Table S2), whose responses are often limited by comparatively slow adsorption–desorption equilibration and disordered ion transport pathways. Here, the signal is generated directly from moisture-triggered hydrovoltaic activation, enabling simultaneous self-powered operation and rapid response.

Another important implication of this work is that sensing behavior can be tuned not only by the internal pore structure but also by the macroscopic fiber configuration. The different performances of linear, helical, and branched devices indicate that device geometry governs the balance between sensing range, transport directionality, and response speed. In particular, the branched architecture provides multiple moisture collection and transport pathways, likely reducing the effective transport distance and improving response efficiency at distributed monitoring points. This multiscale tunability—from nanoscale pore reconstruction to macroscale device assembly—offers a useful strategy for adapting hydrovoltaic sensors to different practical scenarios.

In summary, we have developed porosity-engineered cellulose aerogel fibers that achieve millisecond and highly sensitive hydrovoltaic moisture sensing by fundamentally optimizing multiscale water–ion transport pathways within regenerated biomass microchannels. The markedly enhanced sensing performance originates from (a) the formation of a continuously aligned and hierarchically interconnected nanofiber network that accelerates moisture permeation and enlarges the solid–liquid interfacial area and (b) strengthened ion–water coupling and rapid EDL formation enabled by reduced flow resistance and stabilized ionic distributions within the reconstructed nanochannels. Experimental results, combined with simulation analyses, further reveal that the regenerated porous structure suppresses bubble-induced transport interruptions, supports directional ion migration, and enables fast hydrovoltaic activation under trace moisture inputs, generating detectable voltage within only 70 ms and maintaining a stable output over extended durations. Moreover, the aerogel fibers exhibit multisensory responsiveness to humidity, airflow, temperature, and light, and their performance can be flexibly tuned through distinct structural configurations, including linear, helical, and branched geometries. All 3 configurations achieve rapid responses reaching approximately 0.1 V within 70 to 310 ms and further increase the voltage to 0.49 to 0.63 V within 3 to 5 s. Overall, this work elucidates the pivotal role of hierarchical pore engineering in regulating ion transport and hydrovoltaic conversion in cellulose aerogel fibers, providing new insights for the development of self-powered moisture-sensing devices for storage, logistics, and broader environmental monitoring applications.

## Materials and Methods

### Chemicals

Rattan was obtained from Zhongteng Rattan Industry Co. Ltd. (China). Sodium hydroxide, hydrogen peroxide, sulfuric acid, formic acid, and sodium chloride were purchased from Shanghai Macklin Biochemical Technology Co. Ltd. (Shanghai, China). Shenzhen Yuanxin Delong Technology Co. Ltd supplied conductive carbon paste.

### Preparation of RF

Rattan stems were sectioned into 8 cm in lengths and immersed in deionized water for 1 h to eliminate water-soluble extractives. Delignification was achieved by treating the segments with a 10% (v/v) peracetic acid solution, prepared by mixing 30% H_2_O_2_ and formic acid (1:1 molar ratio) with 1 wt% H_2_SO_4 _as a catalyst, at 55 °C for 24 h. Subsequently, hemicellulose was removed via reaction with a 3 wt% aqueous NaOH solution at 90 °C for 6 h. The resulting samples were thoroughly rinsed with deionized water and lyophilized to yield the disintegrated RFs. In this study, a single xylem vessel is designated as an RF.

### Preparation of RAF

The disintegrated RFs were immersed in a 6 wt% aqueous NaOH solution precooled to −6°C and maintained at this temperature for 48 h to induce partial dissolution of the cell wall components. Subsequently, the samples were regenerated by washing with deionized water until the pH reached neutrality. During this regeneration process, the dissolved components precipitated, reassembling into a nanofiber network. Finally, the RAFs were obtained following lyophilization.

### Preparation of the RAF bundle

Rattan stems were sectioned into 8 cm in lengths and immersed in boiling water for 1 h. To achieve partial processing, only the lower 6-cm segment of each stem was subjected to the sequential delignification and hemicellulose removal procedures described for RF preparation. Subsequently, these locally disintegrated stems underwent the alkaline dissolution and regeneration steps identical to those used for RAF fabrication, yielding the final RAF bundles.

### Characterization

The morphologies of RF and RAF were characterized using field-emission scanning electron microscopy. To verify water distribution, Raman spectra and 2D Raman mapping were acquired on samples containing trace moisture using a Raman spectrometer (LabRAM Soleil, HORIBA, France). Water transport dynamics within the fibers were visualized via polarizing optical microscopy (Nikon, Japan). Nitrogen adsorption–desorption tests were performed on a Micromeritics ASAP 2460 instrument to determine the specific surface area and pore structure of the samples. Electrical properties, including *I–T* and *V–T* curves, were measured using a Keithley 2400 SourceMeter with electrodes coated in conductive carbon paste. Surface hydrophilicity was assessed by measuring water contact angles using a goniometer (Shanghai Zhongchen Digital Technology Apparatus Co. Ltd., China). Finally, low-field ^1^H NMR spectra were recorded on a VTMR20-010V-I NMR analyzer (Suzhou Niumag Analytical Instrument Corp., China) to investigate the water states.

## Data Availability

The data that support the findings of this study are available from the corresponding authors upon reasonable.
