# Supplementary material for: Bioderived Cellulose Aerogel Fibers with Hierarchical Porosity for Millisecond Hydrovoltaic Sensing
Source: Research (Wash D C). 2026 Jun 9;9:1324. doi: 10.34133/research.1324 (PMC13247317; doi:10.34133/research.1324)
Supplement: Supplementary 1 — Figs. S1 to S33 Tables S1 to S3 Movies S1 to S4 [file research.1324.f1.zip › Supporting Information-Clean version.docx]

Supplementary Materials

**Bio-derived Cellulose Aerogel Fibers with Hierarchical Porosity for Millisecond Hydrovoltaic Sensing**

Lanyue Zhang, Yuqing Xie, Shiang Shi, Xiang Chen, Xiaotong Fu, Qichun Feng, Dongdong Ye*

School of Materials and Chemistry, Anhui Agricultural University, Hefei, Anhui Province 230036, China

*Corresponding to*: ydd@whu.edu.cn* (D. Y.)

Table S1. Comparison of RAF water transport velocity with recently reported materials.

**
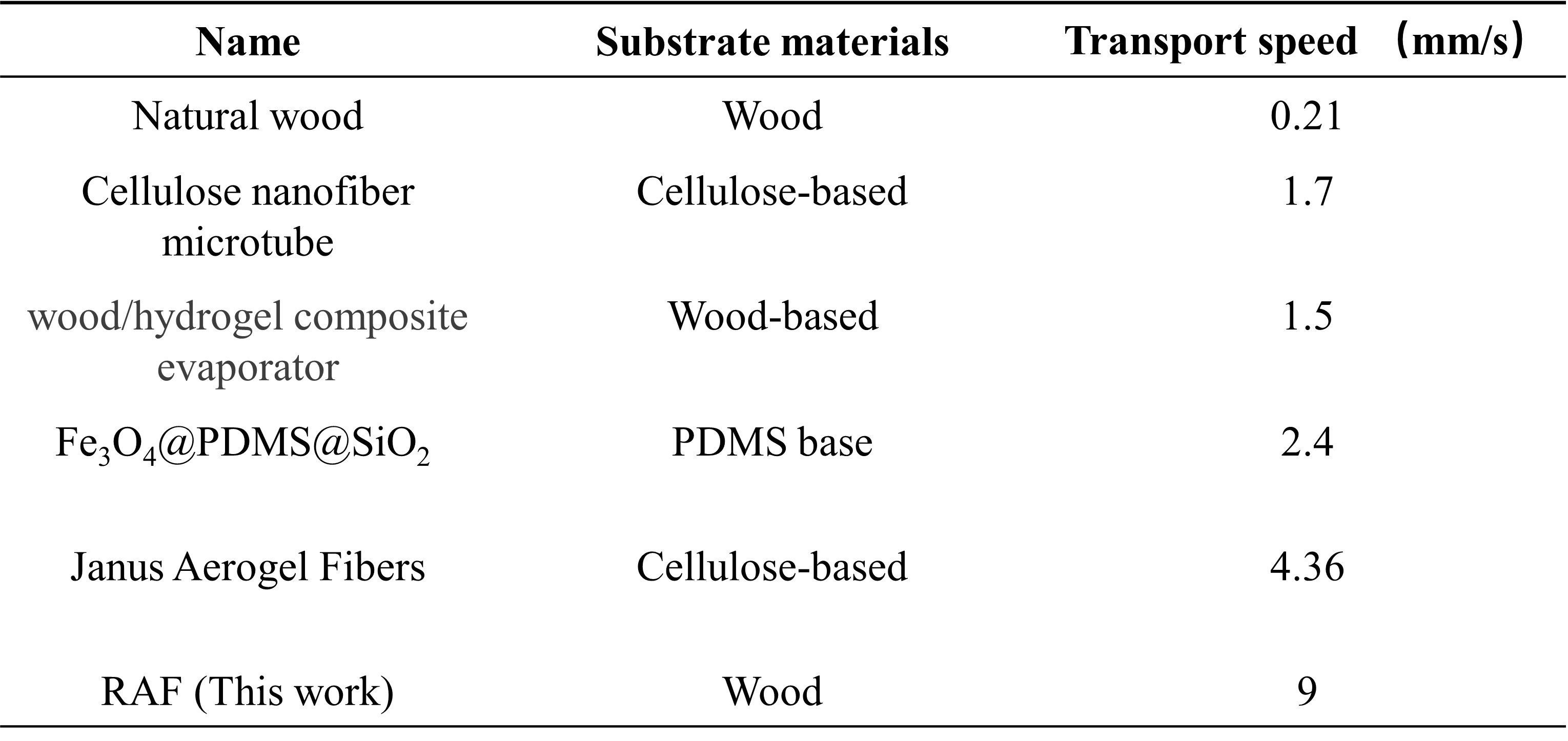
**

Table S2. Comparison of response times among different types of sensors.


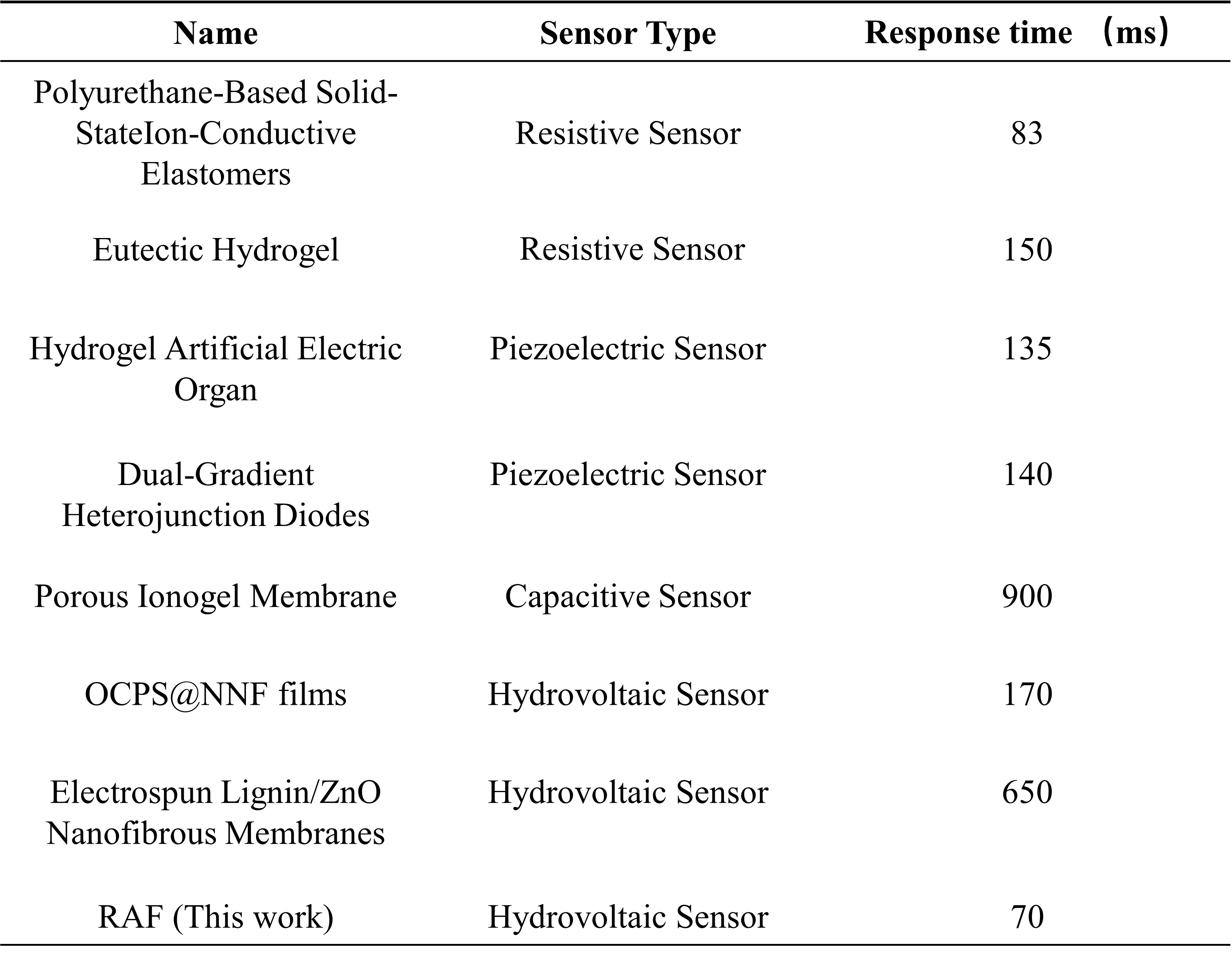


Table S3. Comparison of RAF's response time and open-circuit voltage with recently reported biomass-based hydrovoltaic/moisture-enabled generators.


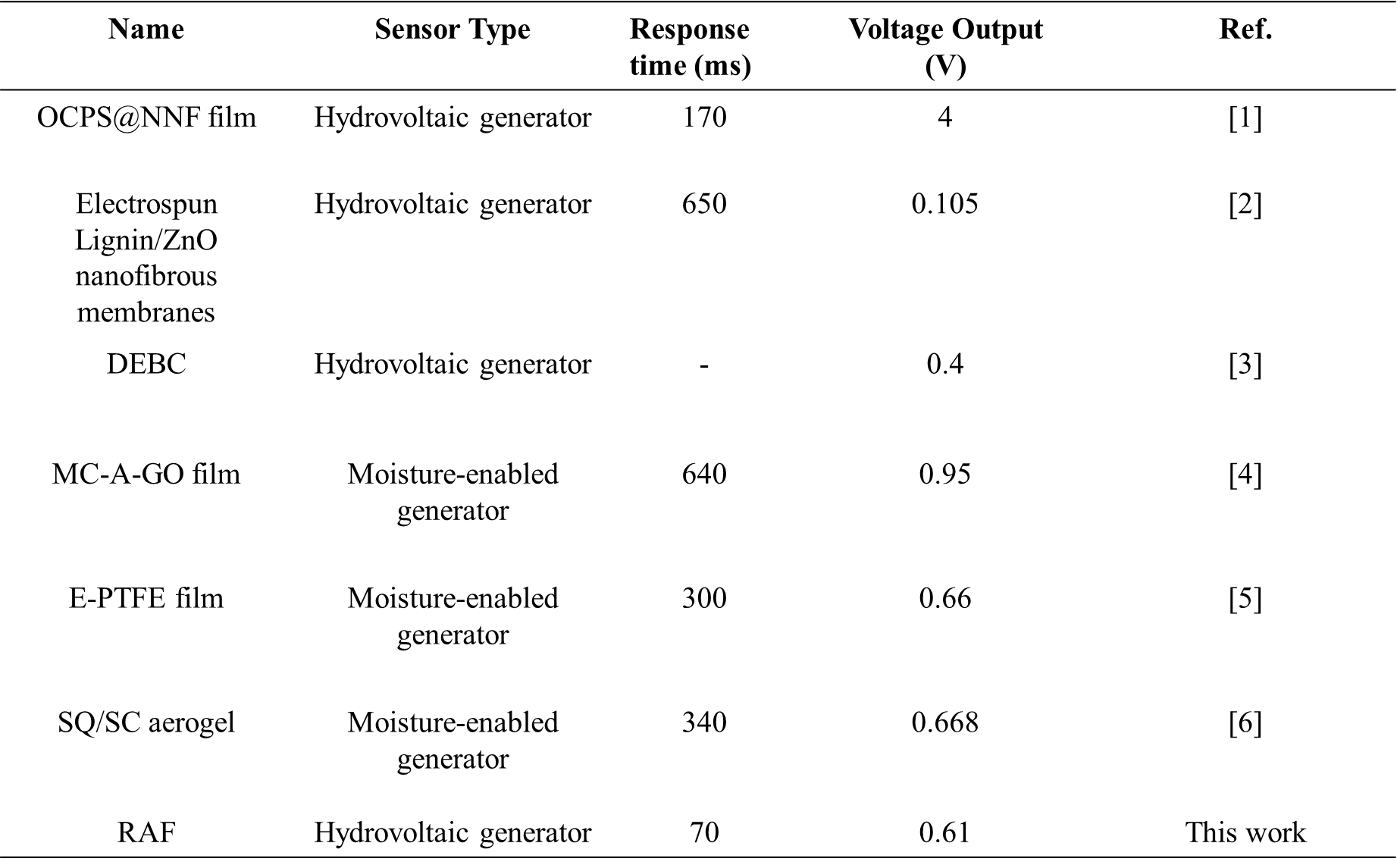


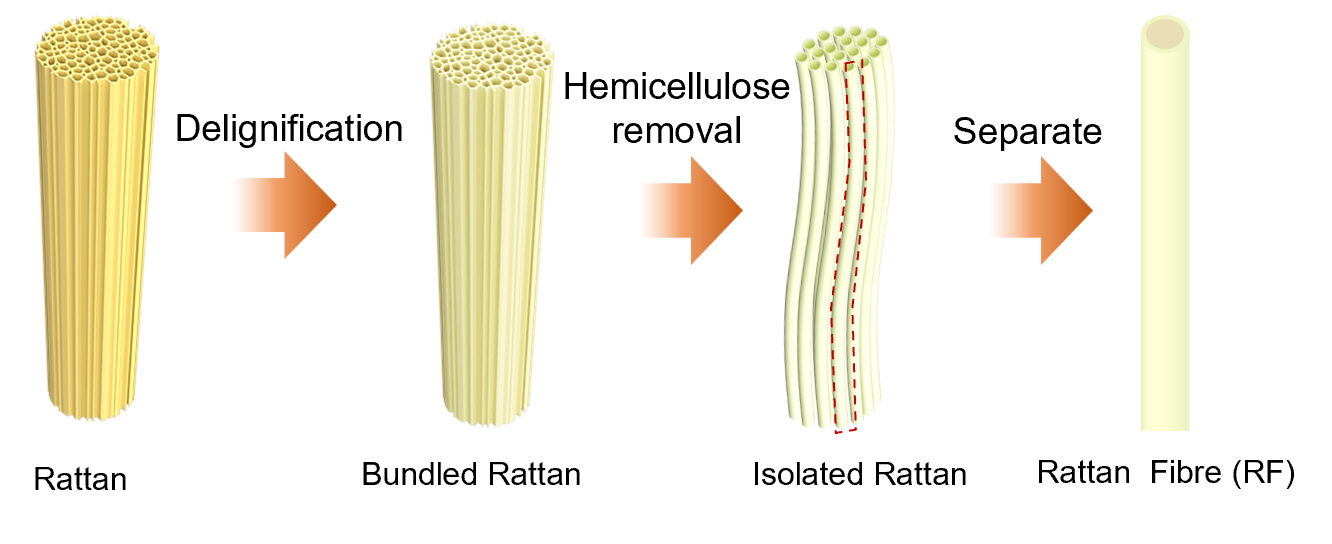


Figure S1. Schematic diagram of the RF preparation process.


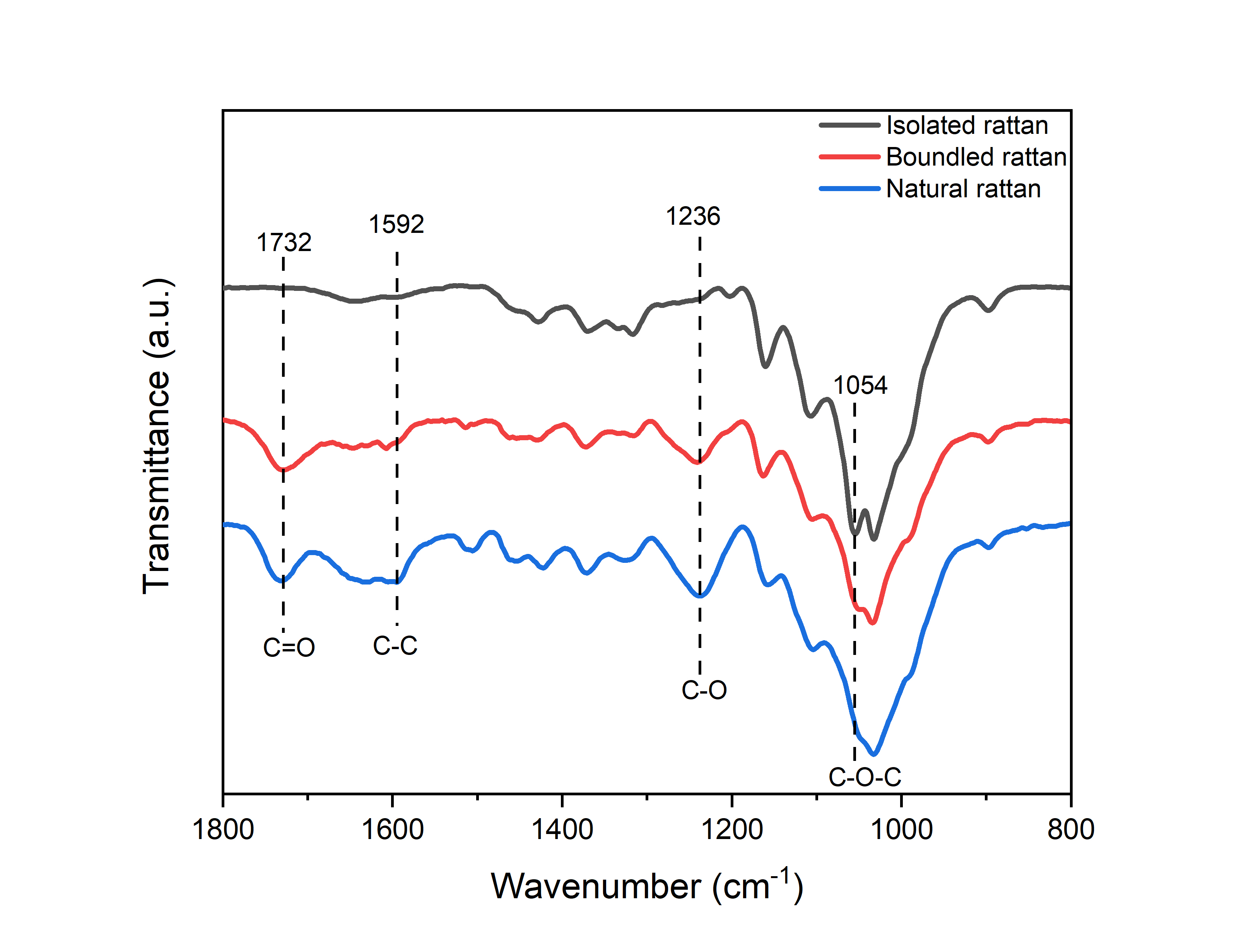


Figure S2. FT-IR spectra of Natural rattan, Boundled rattan, and Isolated rattan.

**
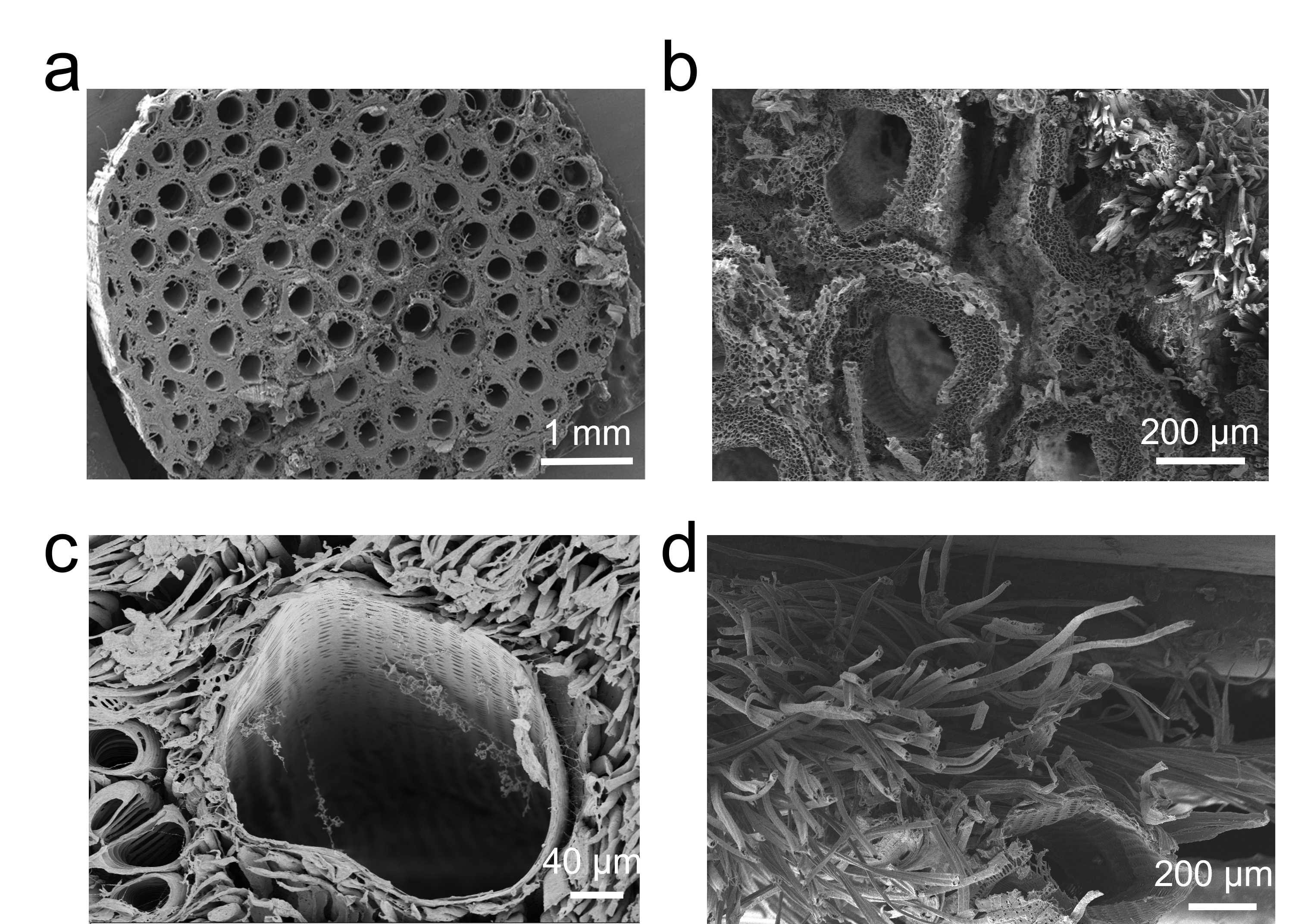
**

Figure S3. SEM images of the rattan. a) The pristine rattan exhibits a dense, porous structure. b) Following delignification, the cell walls progressively separate. c) Subsequent hemicellulose removal renders the cell cavities and cellulose nanofibrils independent. d) The cell cavities and cellulose nanofibrils achieve complete dispersion.


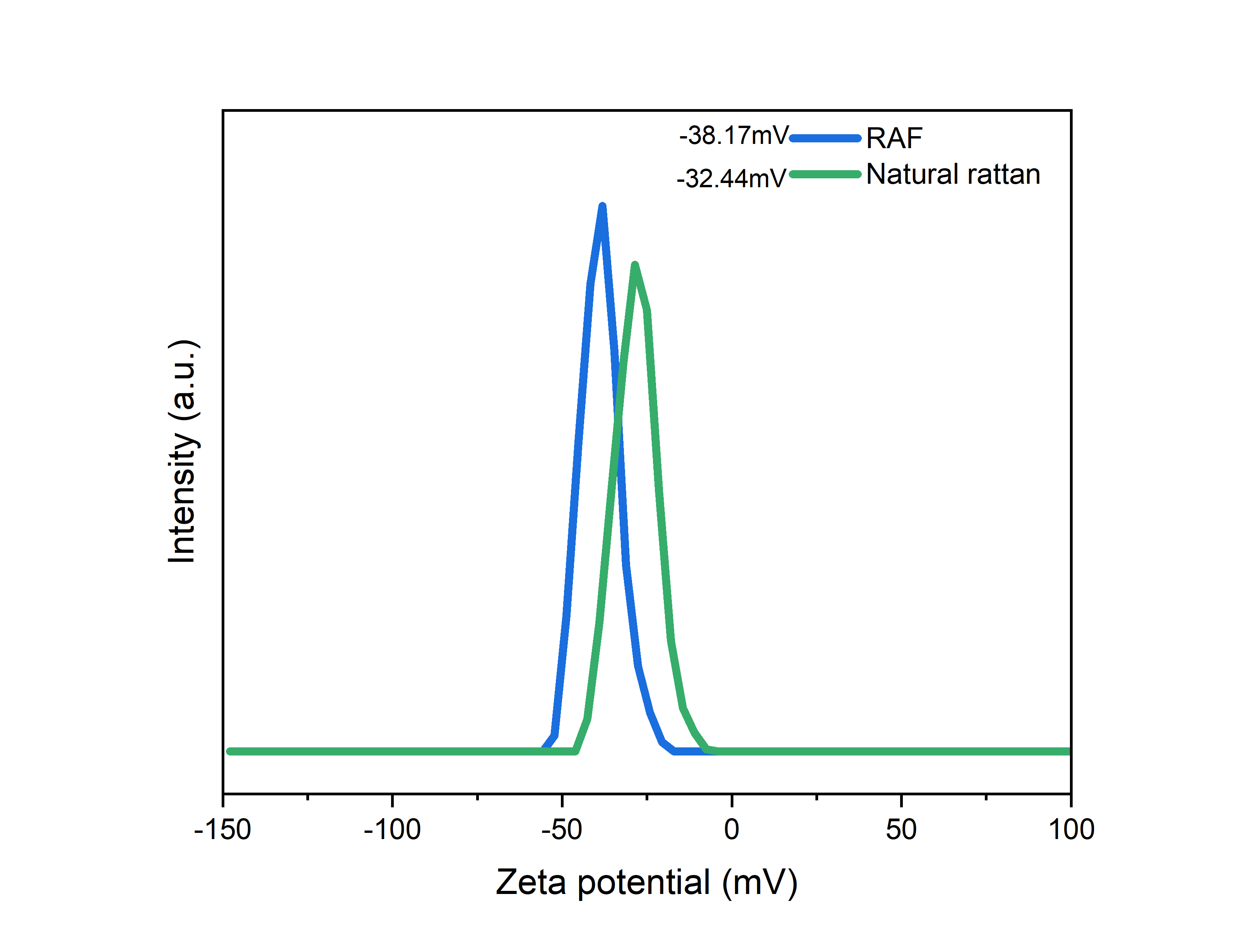


Figure S4. Zeta potential of natural rattan and RAF.


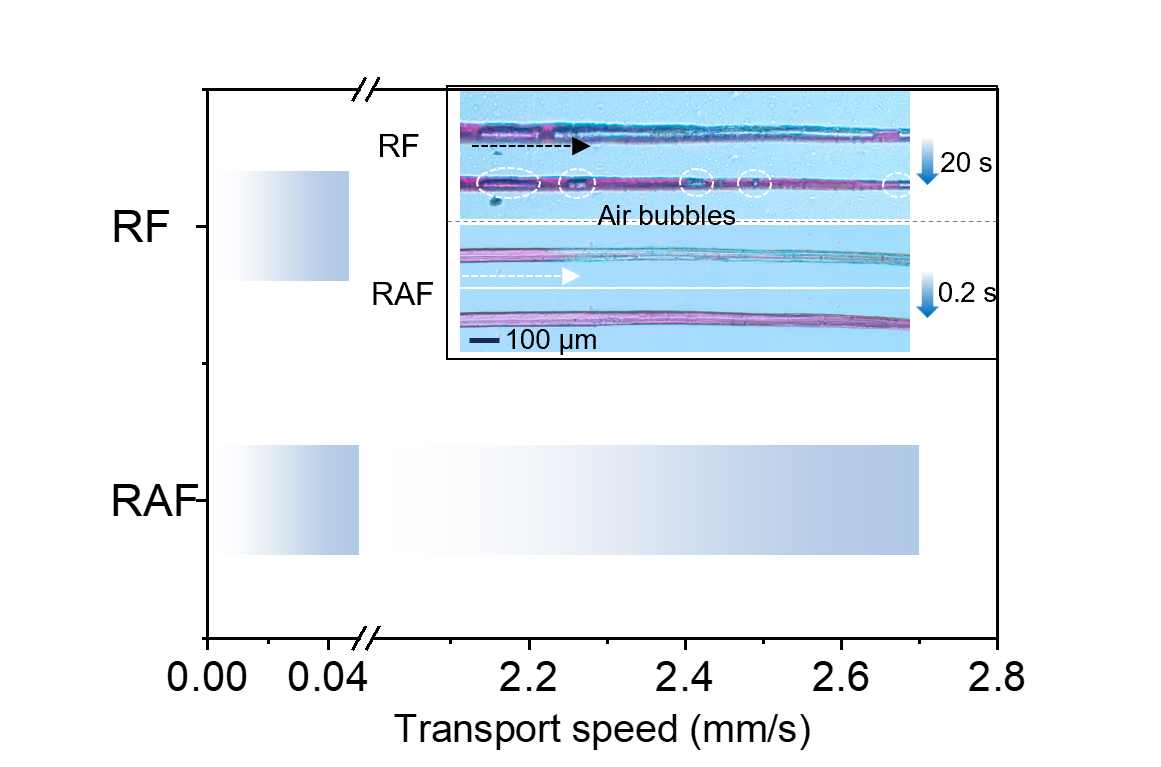


Figure S5. Water transmission speeds of RF and RAF, and digital photos.

- **Behaviors in Native Fibers (Prone to cavitation & signal interruption):**
  - Wall-guided transport: When a native conduit is filled with bubbles or cavities, it prevents the formation of a continuous water column. Under this condition, the continuous nanochannels (composed of spiral nanofibers) on the conduit wall can generate radial negative pressure via a “spiral Venturi effect.” This allows water to rise steadily along the wall in a nearly frictionless state, meaning water is transported along the wall rather than through the main lumen.
  - Bidirectional xylem flow: The bubbles occupy the lumen space and disrupt the continuous axial negative pressure drive. When water transporting along the wall re-enters the lumen, it becomes confined by bubbles at both ends, forming restricted water segments. These segments cannot achieve directional upward movement and instead undergo chaotic, bidirectional diffusive flow, leading to unstable electrical output.
- **Behaviors in Regenerated Cellulose Aerogel Fibers (Cavitation-resistant & stable output performance):**
  - Omnidirectional flow: The regenerated 3D cellulose nanonetwork effectively fills the lumen. It breaks the limitation of single-direction axial transport found in native conduits. This highly interconnected network allows water molecules to rapidly diffuse and permeate along multiple directions (transverse, longitudinal, and oblique), achieving a more thorough, uniform, and low-resistance water transport pathway.
  - Thermodynamic cavitation resistance: The physical mechanism behind the stable transport in Figure 1 is governed by the structural suppression of bubbles, mathematically explained by two key equations.

First, according to the **Young-Laplace equation**:

$P_{c}=\frac{2\gamma\cos\theta}{r}$ (1)

where *P*_c_ is the capillary pressure, *γ* is the surface tension of water, *θ* is the solid–liquid contact angle, and r is the pore radius. The 3D nanofibril network divides the original large vessels into abundant nanoscale channels, drastically reducing r*r* and exponentially enhancing *P_c_*. This strengthened capillary effect enables rapid, full infiltration, eliminating stable gas-liquid interfaces.

Second, according to the **critical bubble nucleation equation**:

$r_{c}=\frac{2\gamma}{(P_{in}-P_{out})}$ (2)

where *r*_c_ is the critical nucleation radius of a bubble, *P*_in_ is the internal pressure of the bubble, and *P*_out_ is the external liquid pressure. The high capillary pressure introduced by the nanofibril network evidently increases the external liquid pressure *P*_out_, which further raises the critical nucleation radius required for stable bubble formation. Consequently, tiny gas nuclei are thermodynamically suppressed before they can grow and block the transport pathways.


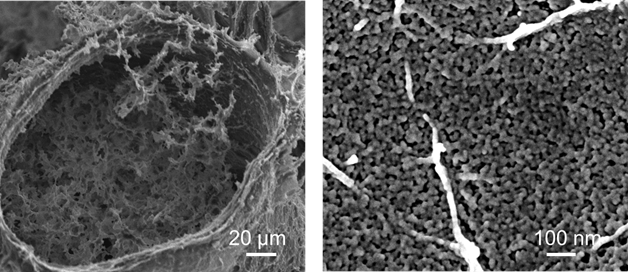


Figure S6. SEM images of the regenerated cellulose nanofiber network inside RAF and the RAF tube wall.

The cellulose aerogel fibers possess a well-defined hierarchical porous structure: the macropore structure (approximately 190 μm) originates from the retention of the native wood lumen, constituting the main mass transport channel; the tube wall (approximately 1–2 μm), formed by the loosening of the cell wall structure during alkali treatment and regeneration, significantly enhances the pore connectivity of the material; and the mesoporous structure (approximately 3–4 nm) arises from the interstices formed by the regenerated cellulose nanofiber network within the lumen, providing a high specific surface area.

**
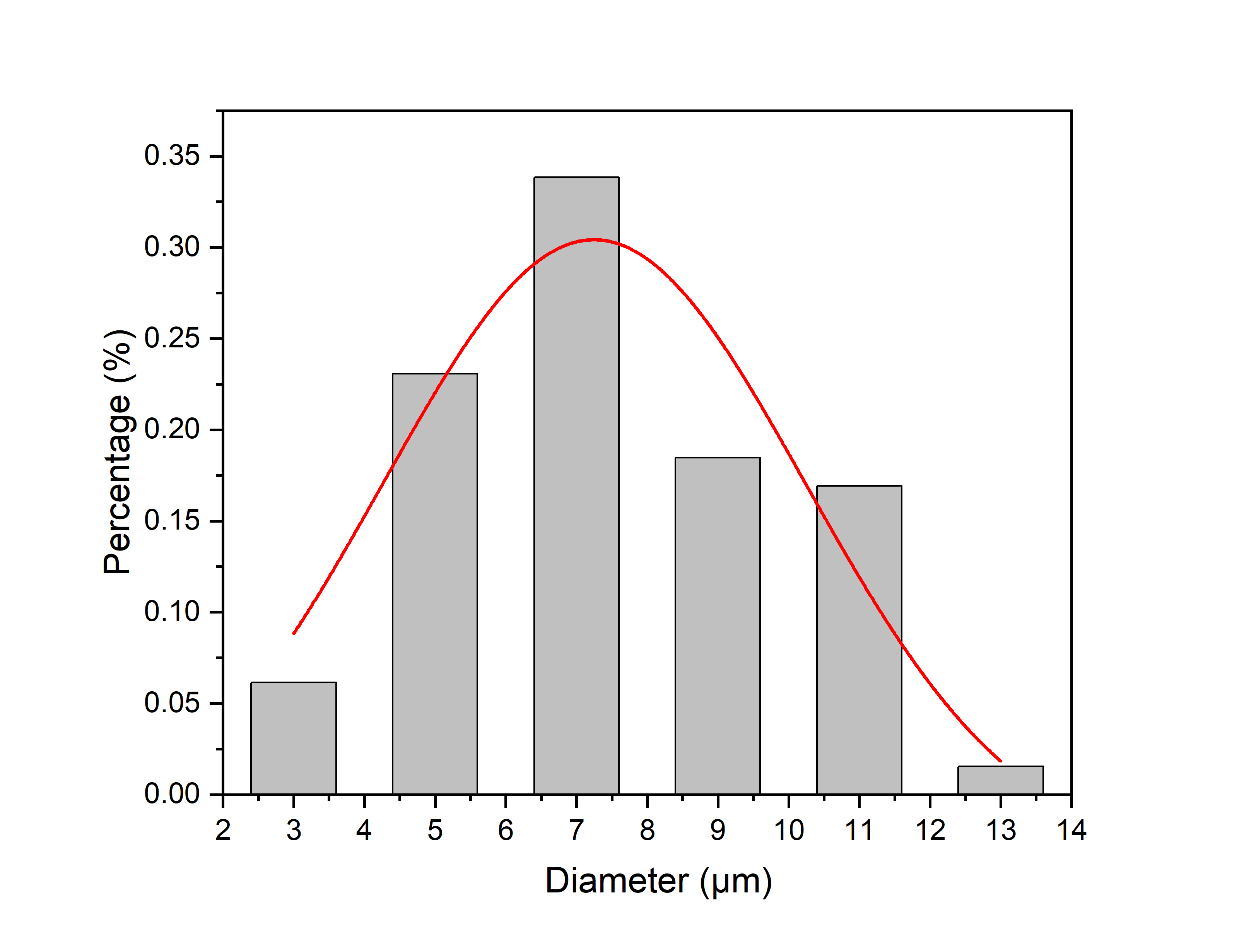
**

**Figure S7.** Particle size distribution of RAF.


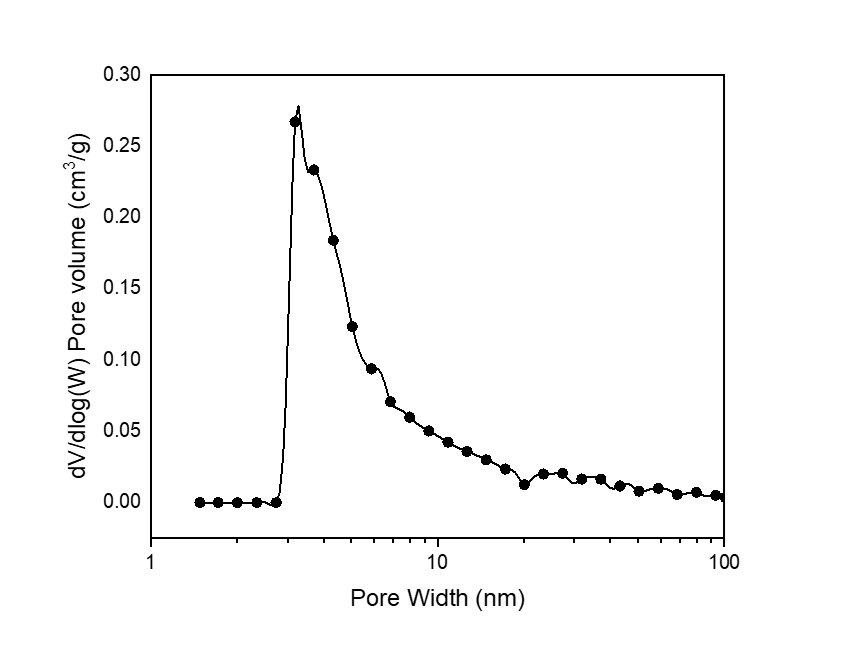


**Figure S8.** **Physisorption pore-size distribution of RAF.**

Nitrogen adsorption–desorption measurements were performed on a Micromeritics ASAP 2460 instrument at 77.30 K to characterize the specific surface area and pore structure of the sample. Before measurement, the sample was vacuum degassed in advance to remove adsorbed moisture and residual impurities. The adsorption and desorption isotherms were collected over a relative pressure range of 0.05–1.0 P/P_0_ under liquid nitrogen atmosphere (−196 °C). The Brunauer–Emmett–Teller (BET) model was adopted to calculate the specific surface area within the relative pressure range of 0.05–0.25 P/P_0_^[7]^. The pore size distribution and cumulative pore volume were analyzed via the Barret–Joyner–Halenda (BJH) model based on the desorption branch of the isotherm.


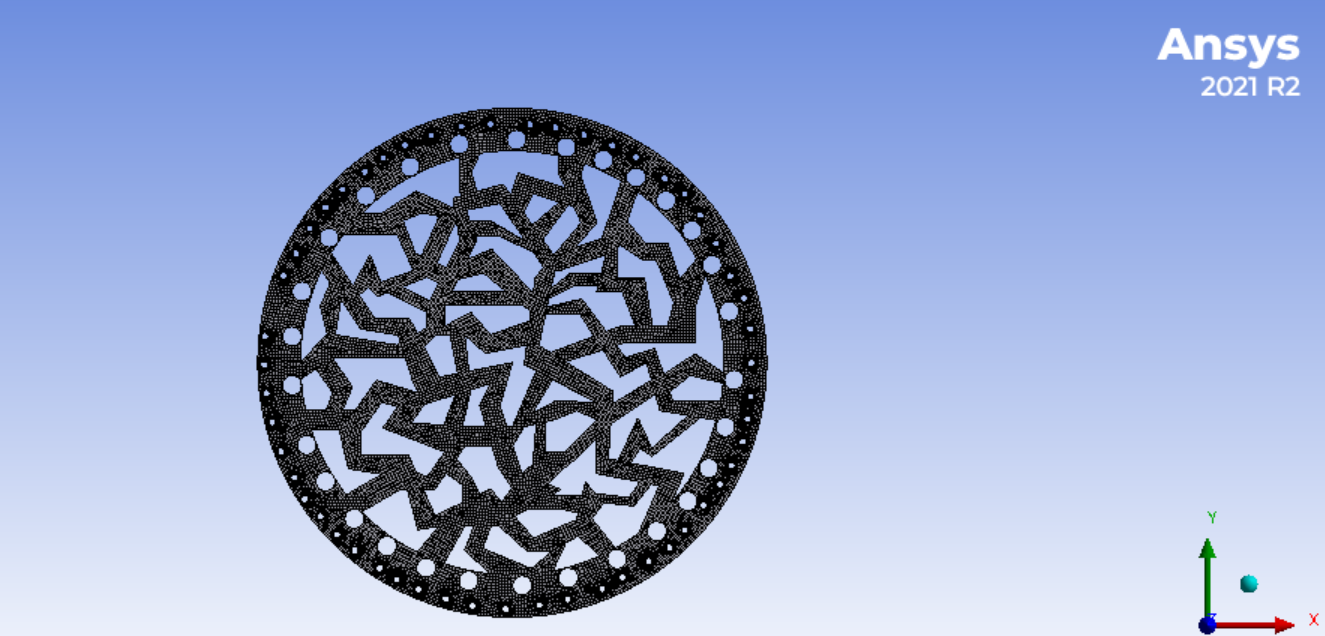


**Figure S9. Schematic diagram illustrating the mesh generation of the RAF model.**

**Geometry generation (porous structure):**

The 2D porous-structure geometry was constructed in ANSYS SpaceClaim 2021 R2. The computational domain is a circular plane with a 40 mm diameter.

1. In the central region, a bio-inspired branching pore network was created using a parametric sketch workflow, including recursive branching and trimming operations to form a fully connected irregular pore-channel network, ensuring uninterrupted flow paths.
2. In the outer annular region, uniform circular micropores were inserted at preset circumferential positions. The micropore diameter is 3 mm, and their locations follow an even distribution along the ring.
   Finally, all pore features were subtracted from the solid matrix to obtain the porous structure.

**Meshing strategy and mesh independence:**

Meshing was performed using ANSYS Meshing 2021 R2. Considering the complexity of the porous boundaries, we adopted the **Patch Conforming meshing approach** with a quadrilateral-dominated hybrid element scheme to balance mesh quality and boundary conformity. The nominal element size was set to 0.3 mm, with a growth rate of 1.2 and relevance center set to “Fine” to enhance resolution in feature-rich regions. The mesh was checked to avoid negative-volume elements, and the orthogonal quality was > 0.2. To confirm mesh adequacy, a mesh-independence study was conducted using three mesh sizes: 0.2 mm (33,873 elements), 0.3 mm (18,969 elements), and 0.4 mm (13,208 elements; 10,525 elements). The numerical results showed convergence at 0.3 mm; thus, 0.3 mm was selected for all subsequent simulations to ensure accuracy and computational efficiency (**Figure R1** demonstrating the mesh configuration).

**Physics model, assumptions, and numerical settings**:

The numerical simulation was performed using the finite volume method in ANSYS 2021 R2. For velocity–pressure coupling, the **SIMPLEC** algorithm was employed to accelerate convergence, and the **second-order upwind** scheme was applied for the discretization of convection terms. Convergence was achieved when the residual criteria for all variables were below 1×10^-6^, and the energy-equation residual was below 1×10^-8^.

To simplify the simulation while maintaining physical relevance, we adopted the **following assumptions**: i) The flow in the channel is steady, incompressible, continuous, and laminar; ii) Viscous dissipation is neglected; iii) No phase change occurs, and a no-slip condition is applied at the fluid–solid interface. Based on the above assumptions, the governing equations for the numerical simulation can be simplified as follows.

Continuity equation for fluid flow within the channel:

$$\begin{aligned} \frac{\partial u}{\partial x}+\frac{\partial v}{\partial y}=0\#\left( 3 \right) \end{aligned}$$

Momentum equation for fluid flow within the channel:

$$\begin{aligned} \rho_{f}\left( u\frac{\partial u}{\partial x}+v\frac{\partial u}{\partial y} \right)=\mu_{f}\left( \frac{\partial^{2}u}{\partial x^{2}}+\frac{\partial^{2}u}{\partial y^{2}} \right)-\frac{\partial p}{\partial x}\#\left( 4 \right) \end{aligned}$$

$$\begin{aligned} \rho_{f}\left( u\frac{\partial v}{\partial x}+v\frac{\partial v}{\partial y} \right)=\mu_{f}\left( \frac{\partial^{2}v}{\partial x^{2}}+\frac{\partial^{2}v}{\partial y^{2}} \right)-\frac{\partial p}{\partial y}\#\left( 5 \right) \end{aligned}$$

**Boundary conditions:**

(1) Velocity inlet: inlet flow velocity set to 0.2 m/s.

(2) Pressure outlet: pressure outlet prescribed at the domain outlet.

(3) No-slip condition: applied on the fluid–solid interface:

$$\begin{aligned} u_{x}=u_{y}=0\#\left（ 6 \right） \end{aligned}$$

(4) Symmetry boundary conditions: applied on both sides of the computational domain to represent symmetry and reduce computational cost:

$$\begin{aligned} {-k}_{s}\frac{\partial T_{s}}{\partial y}=0\#\left（ 7 \right） \end{aligned}$$

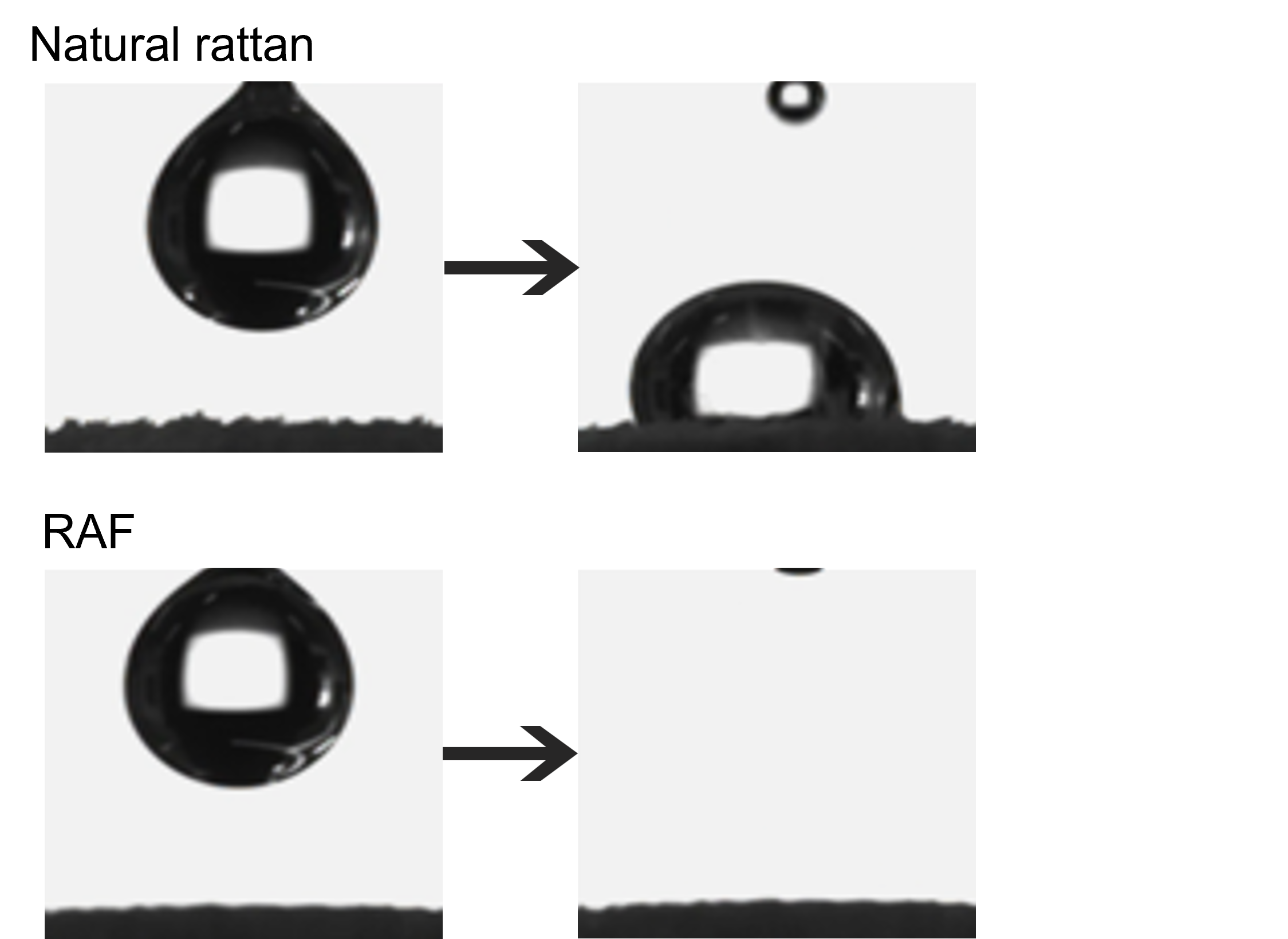


Figure S10. Water contact angle of natural rattan and RAF.

The water contact angle (CA) of the samples was measured using an OCA 40 automatic contact angle goniometer (Data Physics, Germany). A Photron UX100 high-speed camera was employed for droplet impact tests. Owing to the slender morphology of natural rattan fibers, direct contact angle measurement is infeasible. Therefore, the natural rattan was cut into blocks and treated with the same preparation process as natural rattan fibers and RAF to obtain bulk natural rattan and bulk RAF samples. These block samples can be regarded as an aggregate of rattan fibers, and their cross-sections perpendicular to the tube diameter were uniformly selected for contact angle characterization.


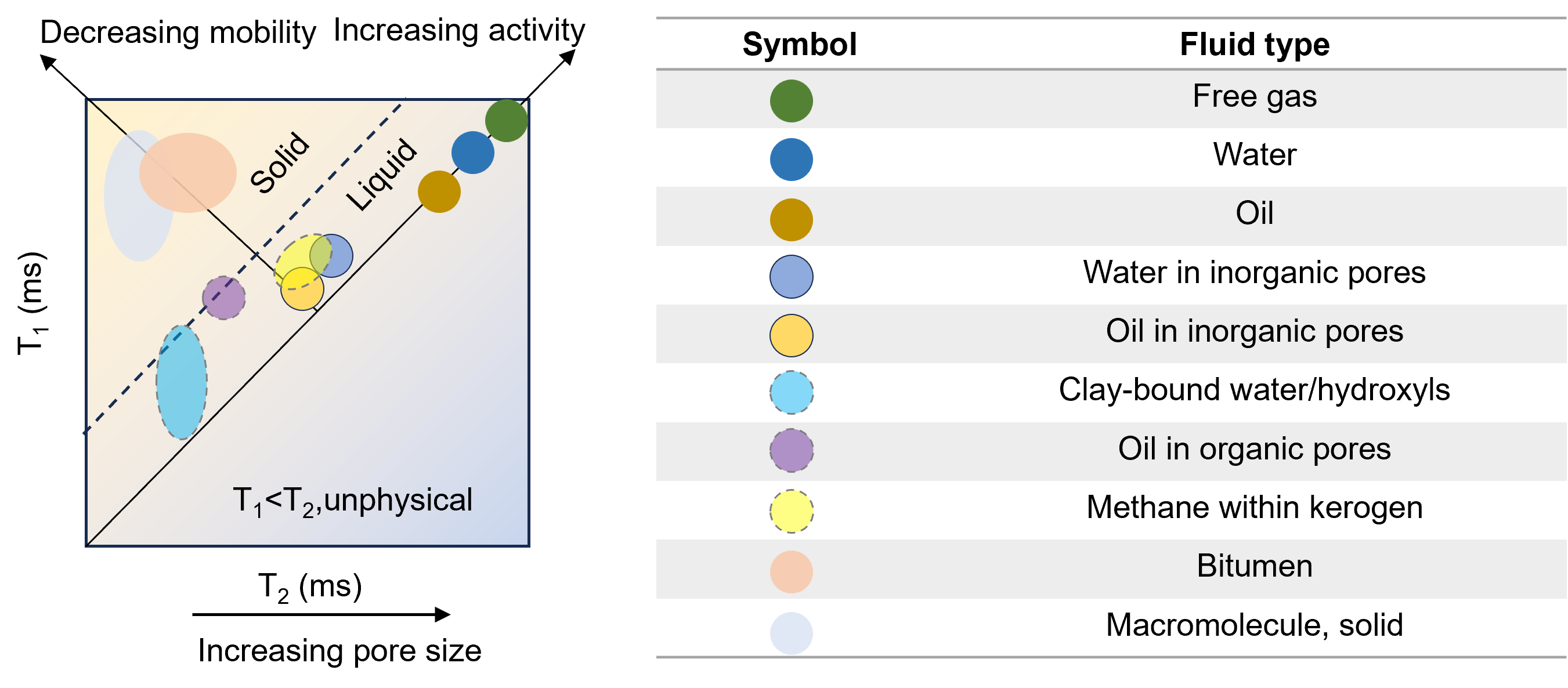


Figure S11. NMR T_1_−T_2_ relaxation map for different fluid types and conditions, adapted from. The short T_1_,_2_ times indicate a small pore structure assigned to bound water. The long T_1_,_2_ times indicate a large pore structure assigned to free water (increasing activity of ^1^H). The large T_1_/T_2_ ratio indicates a significant molecular weight or high-regularity lattice structure assigned to solid matter (decreasing mobility of ^1^H).

- **T_1_** (Longitudinal relaxation time): Characterizes the rate at which water molecules exchange energy with the surrounding solid matrix.
- **T_2_** (Transverse relaxation time): Reflects the mobility and the degree of confinement of the water molecules.
- **T_1_/T_2_** ratio: This ratio serves as a reliable quantitative indicator used in NMR analysis to evaluate the frictional resistance and transport mobility of confined water within hierarchical nanostructures.
  - When T_1_/T_2_ < 10: Water molecules are primarily in a liquid-like state with weak confinement and low friction, enabling highly efficient transport.
  - When T_1_/T_2_ ≥10: The movement of water molecules is significantly restricted. The water behaves similarly to solid-bound water, exhibiting poor fluidity and high transport resistance.


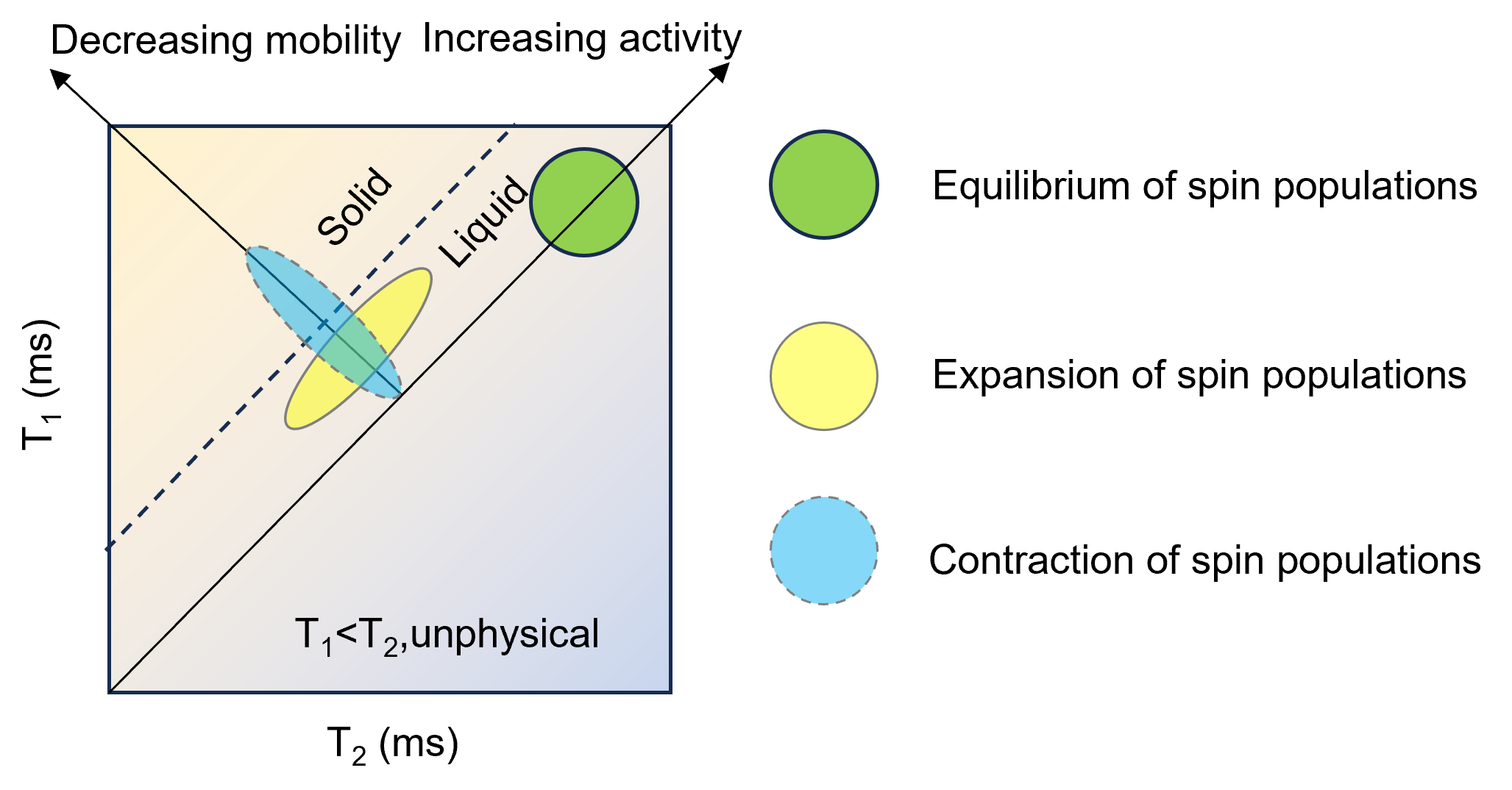


Figure S12. NMR T_1_−T_2_ relaxation map for different fluid states. The circular pattern indicates stable entropy, where ^1^H remains confined and does not exchange with the external environment. The spindle pattern, aligned parallel to the diagonal, represents an open space where 1H exchanges with the external environment, resulting in an increase in entropy. The spindle pattern perpendicular to the diagonal indicates a semi-closed space where ^1^H is constrained by the external environment, resulting in a decrease in entropy.


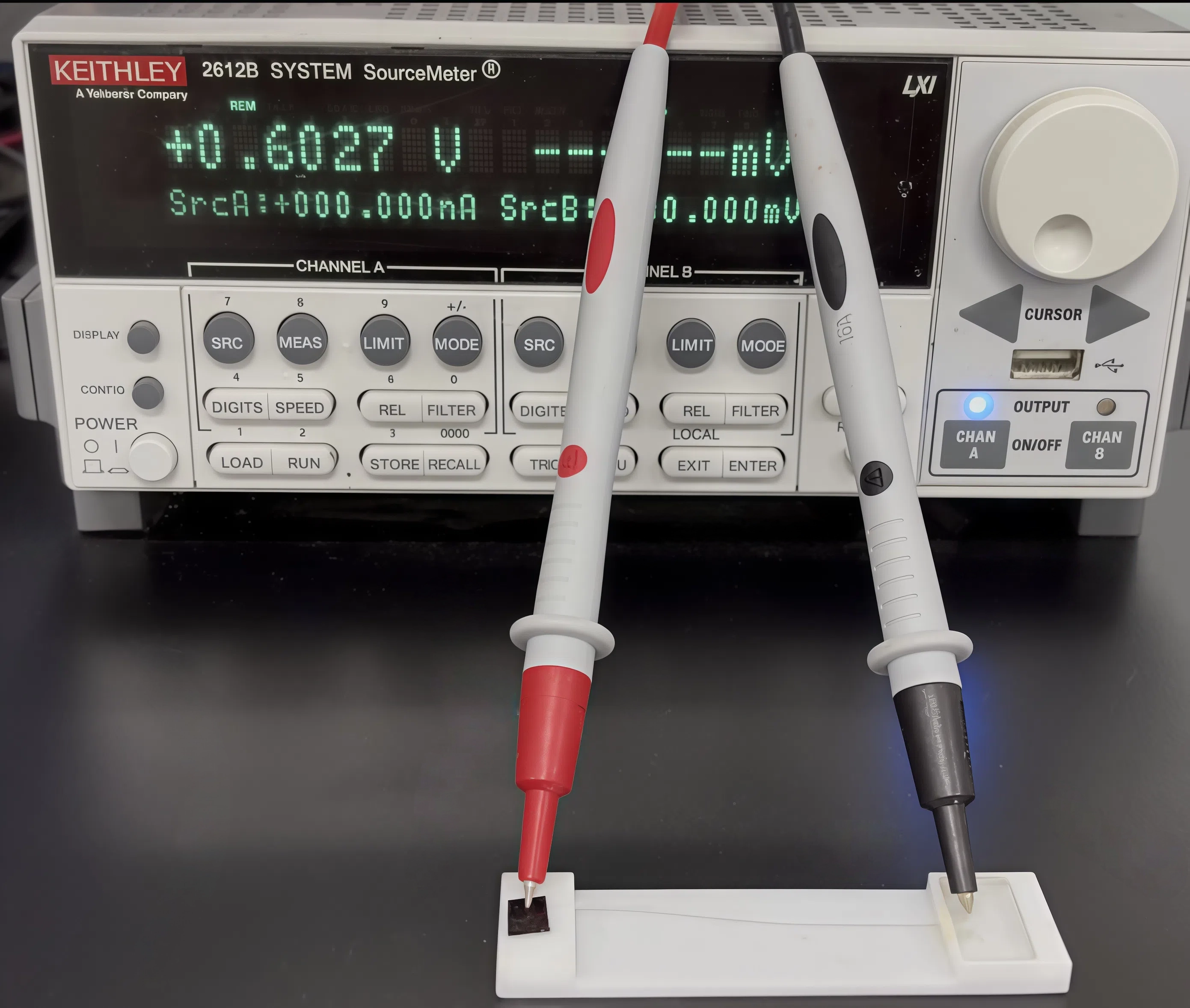


Figure S13. Figures of the power generation device.


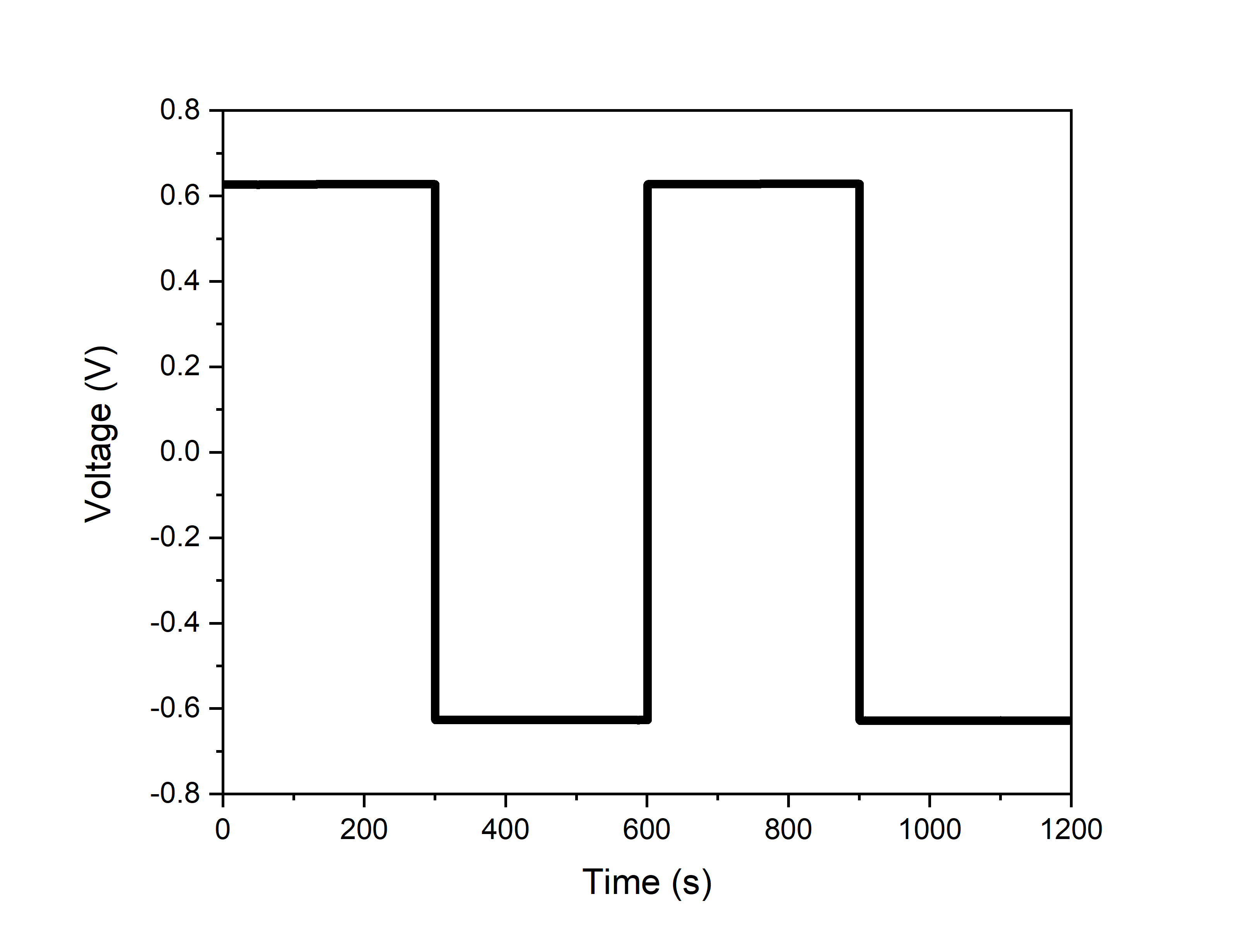


Figure S14. Output voltage of the RAF before and after polarity conversion.


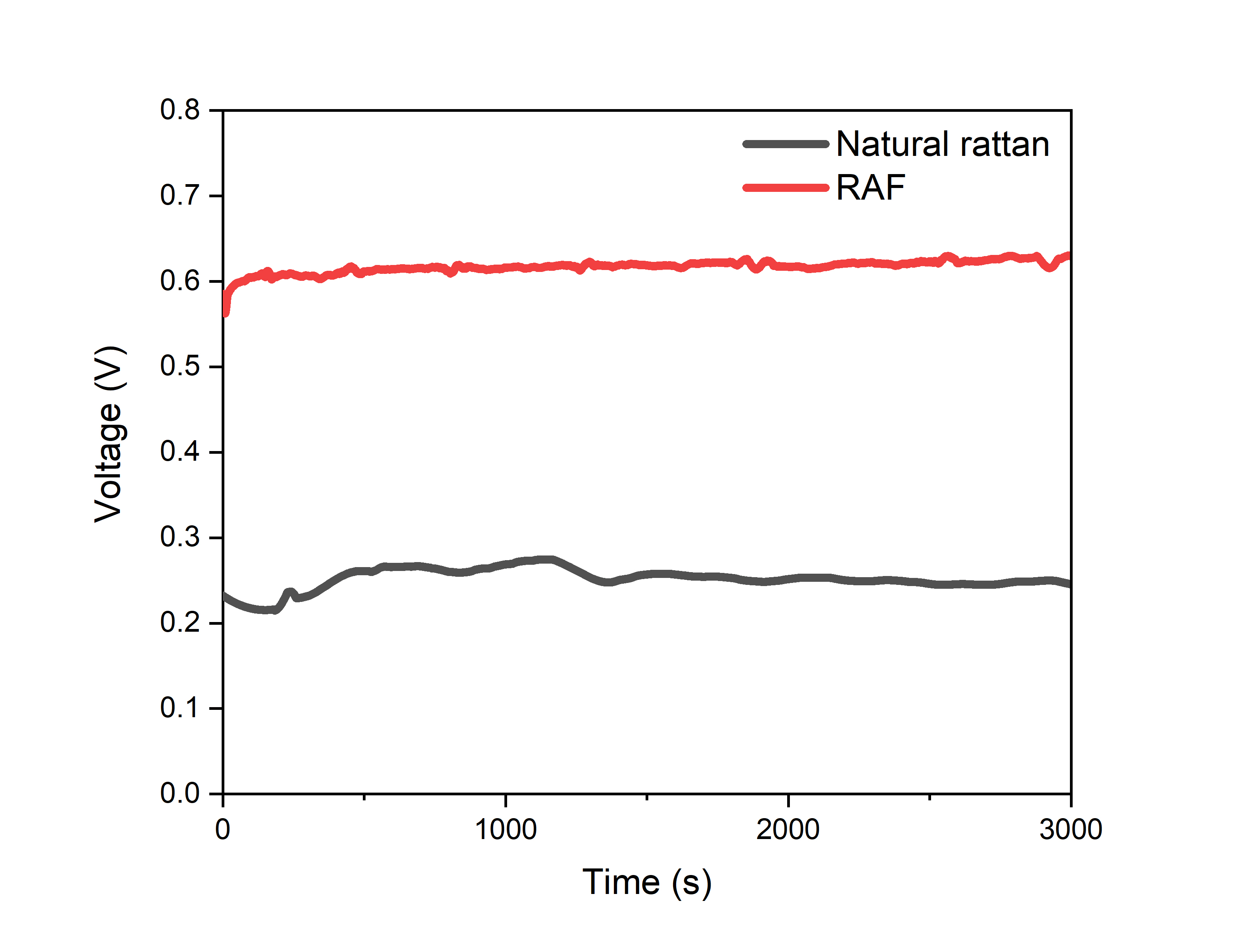


Figure S15. Output voltage curves for natural rattan and RAF under 2.5 wt% NaCl solution conditions.


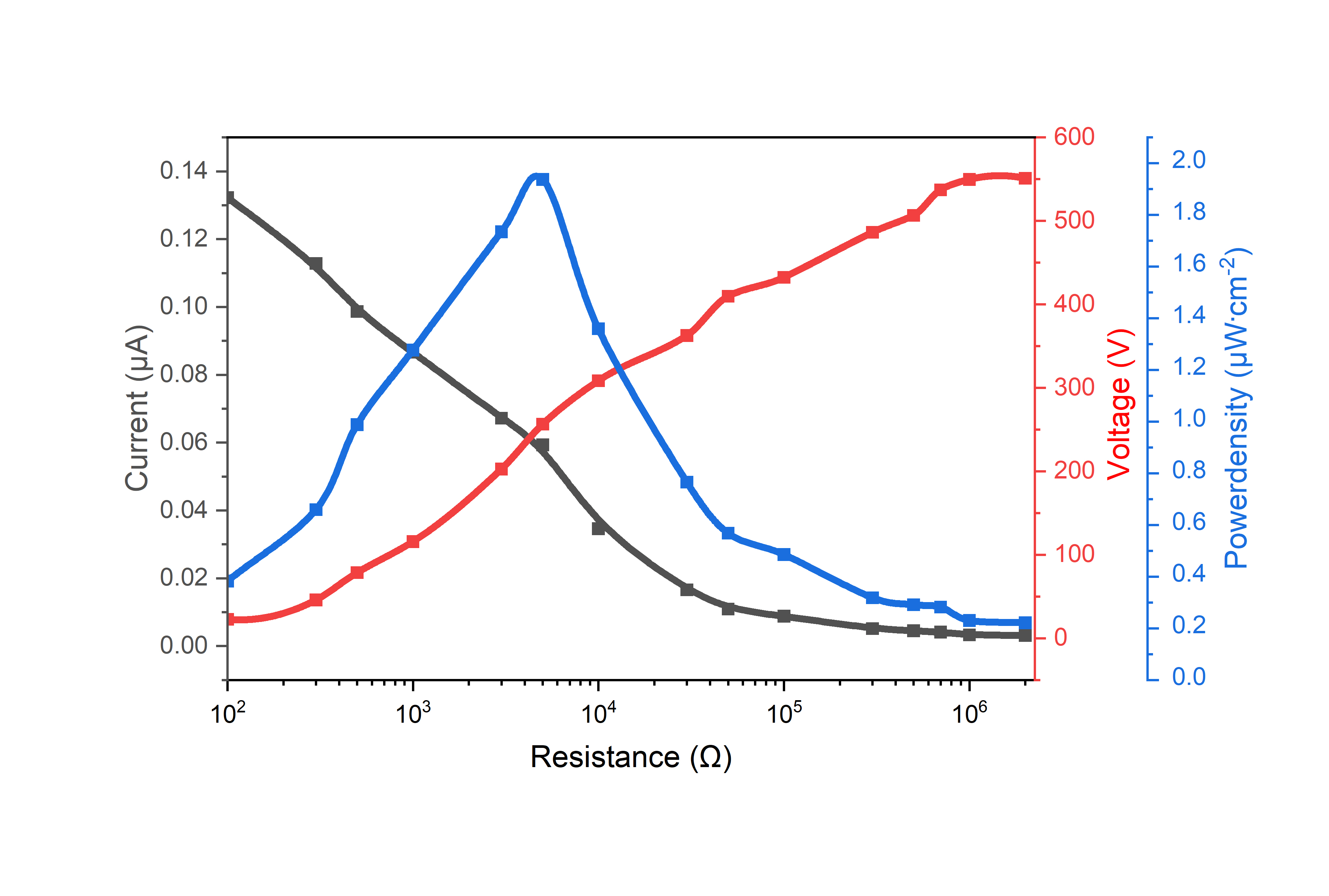


Figure S16. The open-circuit voltage, short-circuit current, and power density of RAF under different load resistance.


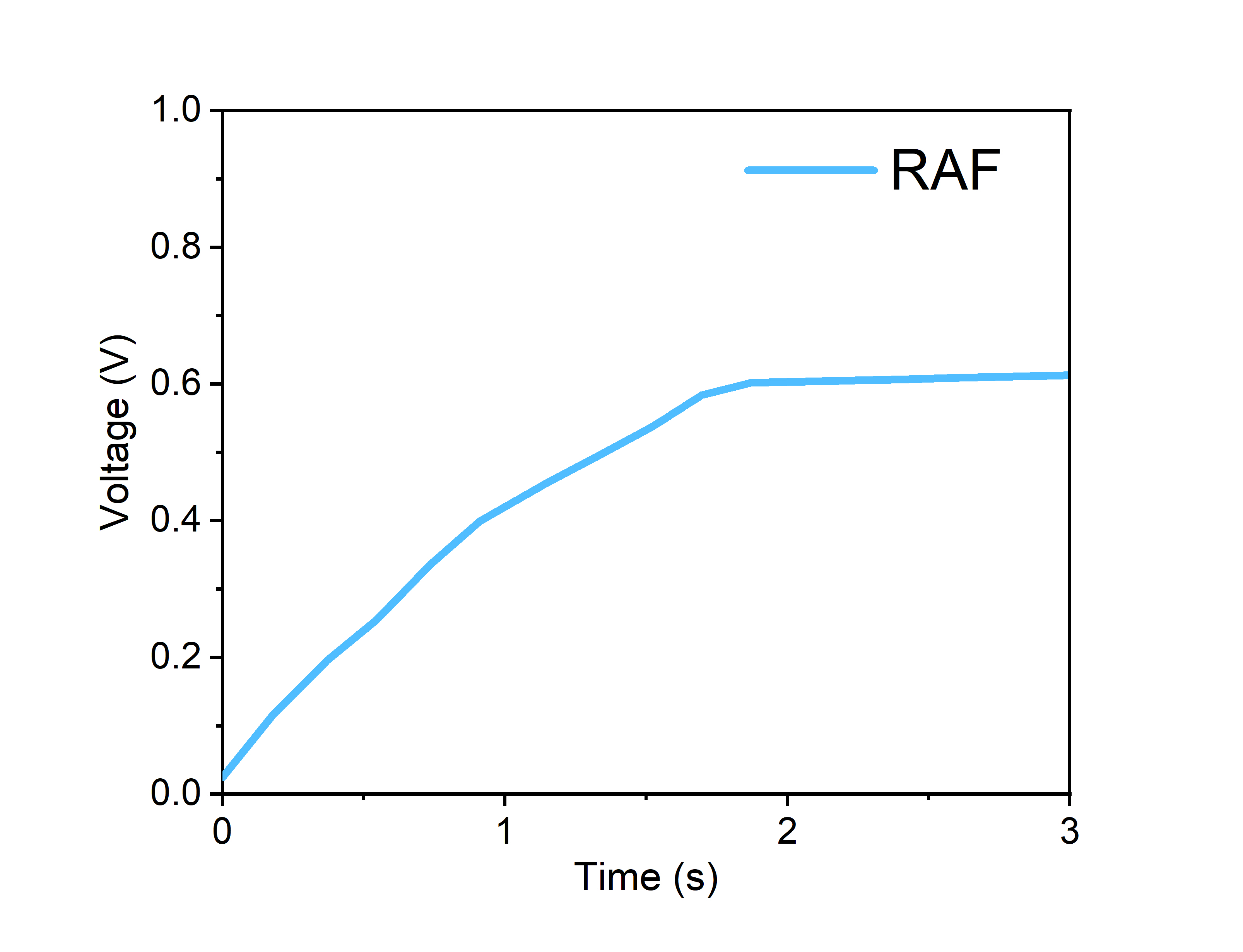


Figure S17. The enlarged view of the V_OC_ signal from RAF after adding 10 μL of 2.5 wt% NaCl solution.


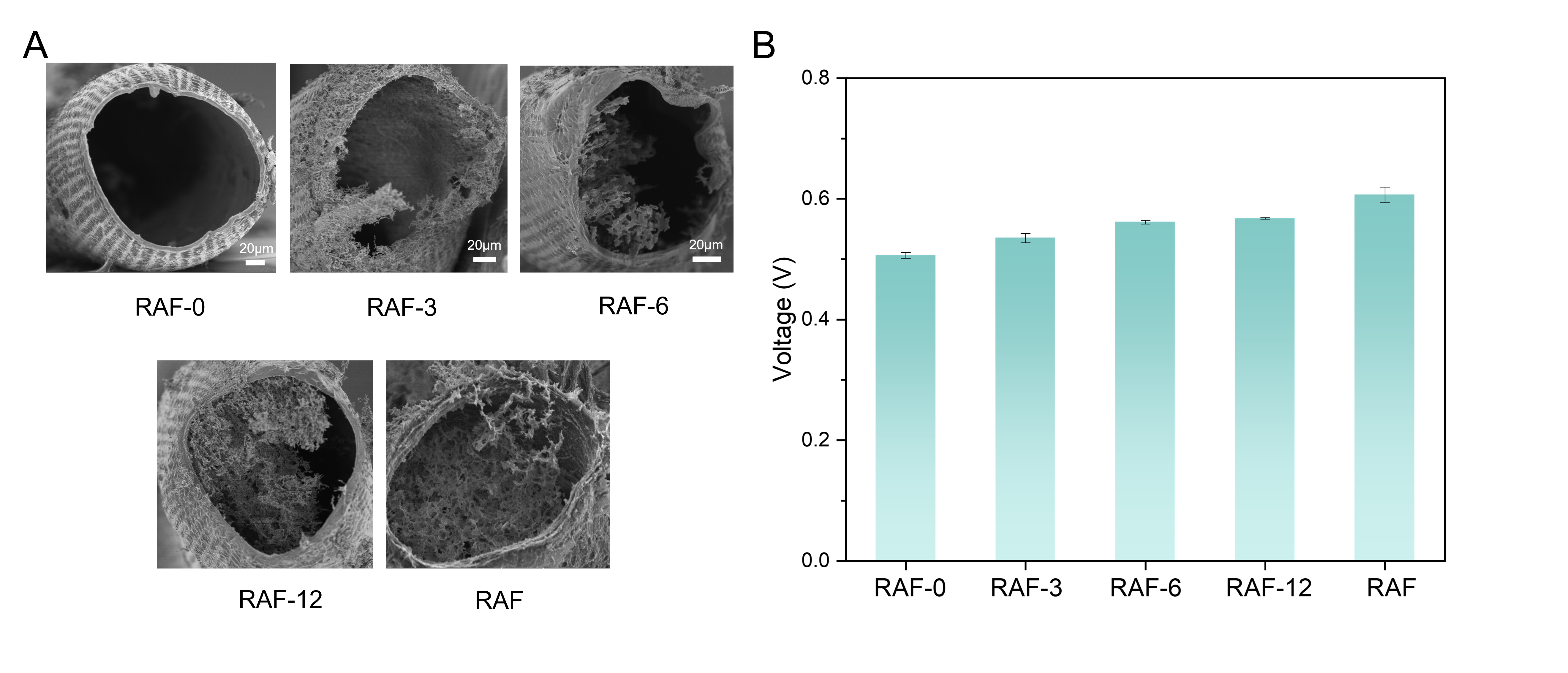


Figure S18. a) Cross-sectional scanning electron microscopy (SEM) images of regenerated cellulose network-based RAF fibers with different regeneration degrees, denoted as RAF-0, RAF-3, RAF-6, RAF-12, and RAF, respectively. b) Output voltage of RAF at different regeneration degrees.


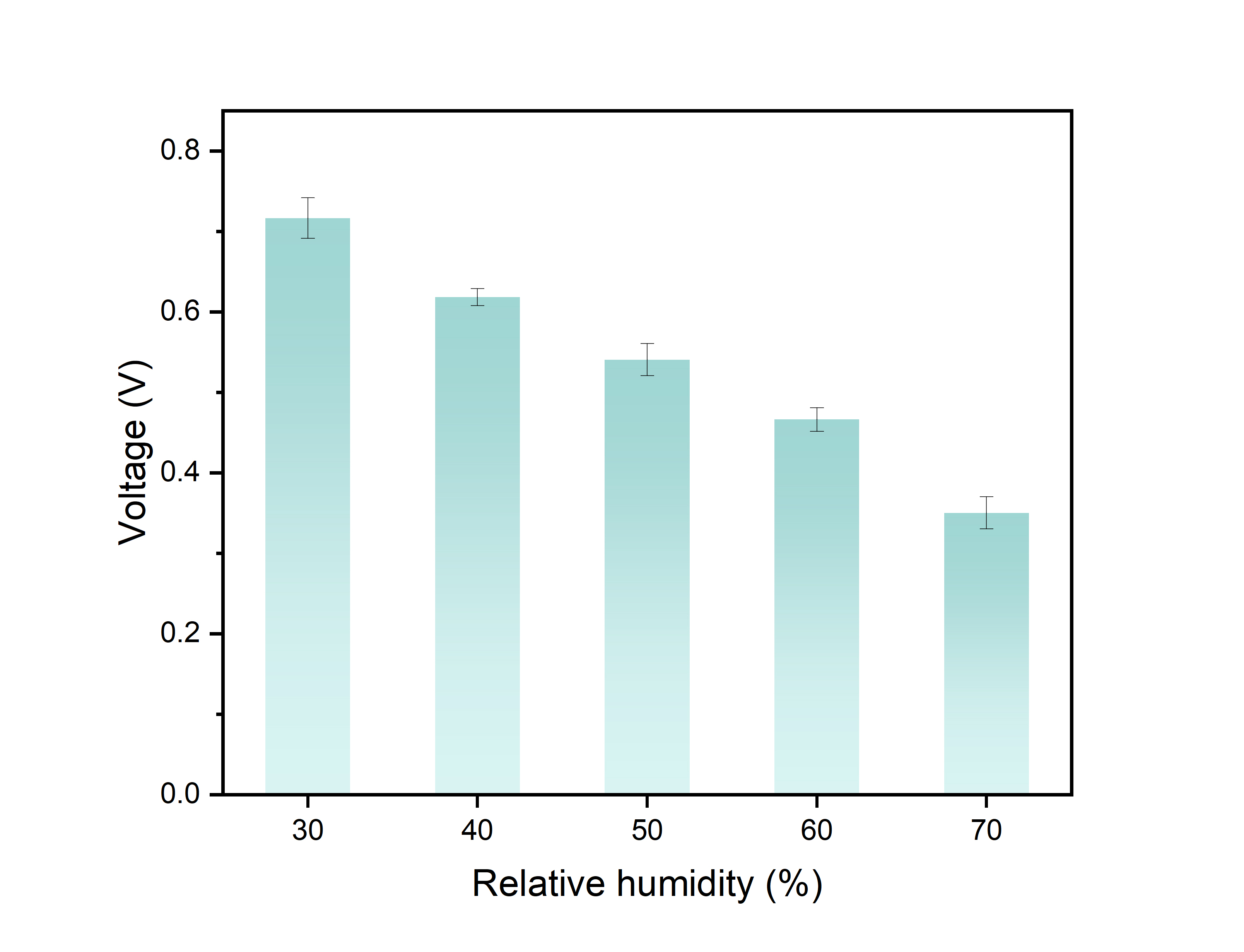


Figure S19. Output voltage of RAF at different humidity levels.


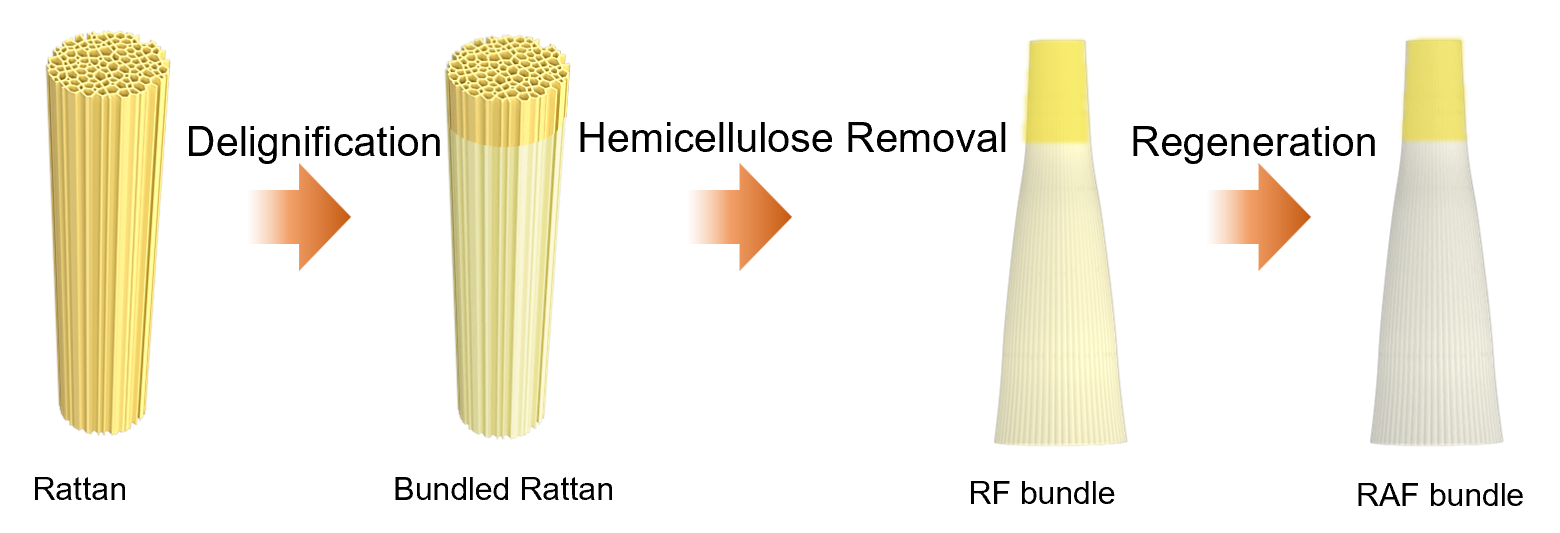


Figure S20. Schematic diagram of the RAF bundle preparation process.


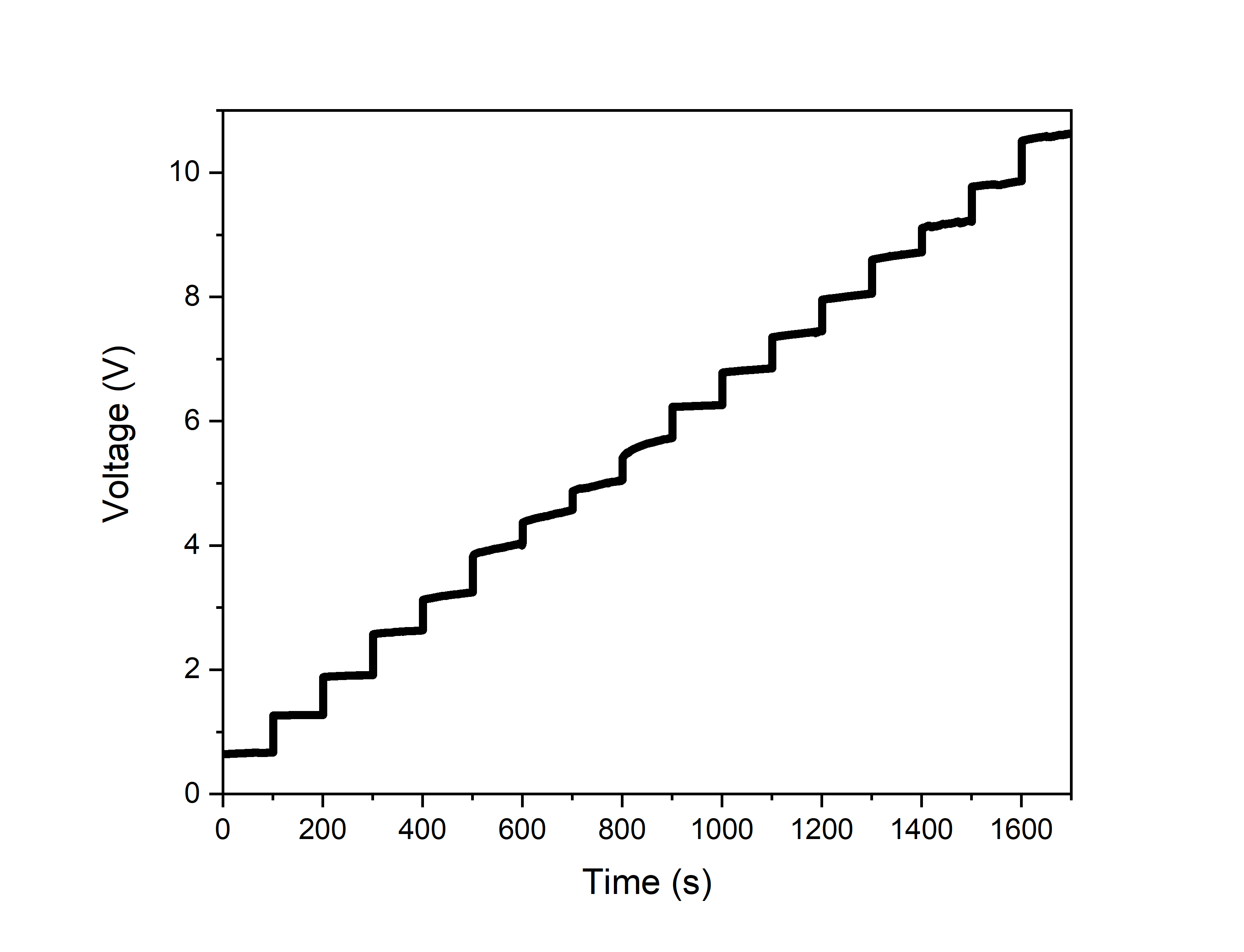


Figure S21. Voltage output from 17 series-connected RAFs.

**
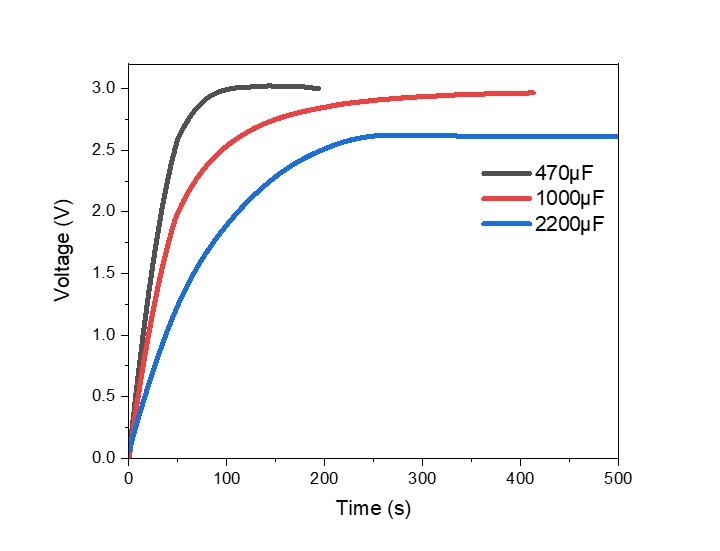
**

**Figure S22. Voltage–time profiles of commercial capacitors (470, 1000, and 2200 μF) charged by the integrated aerogel fibers.**

**
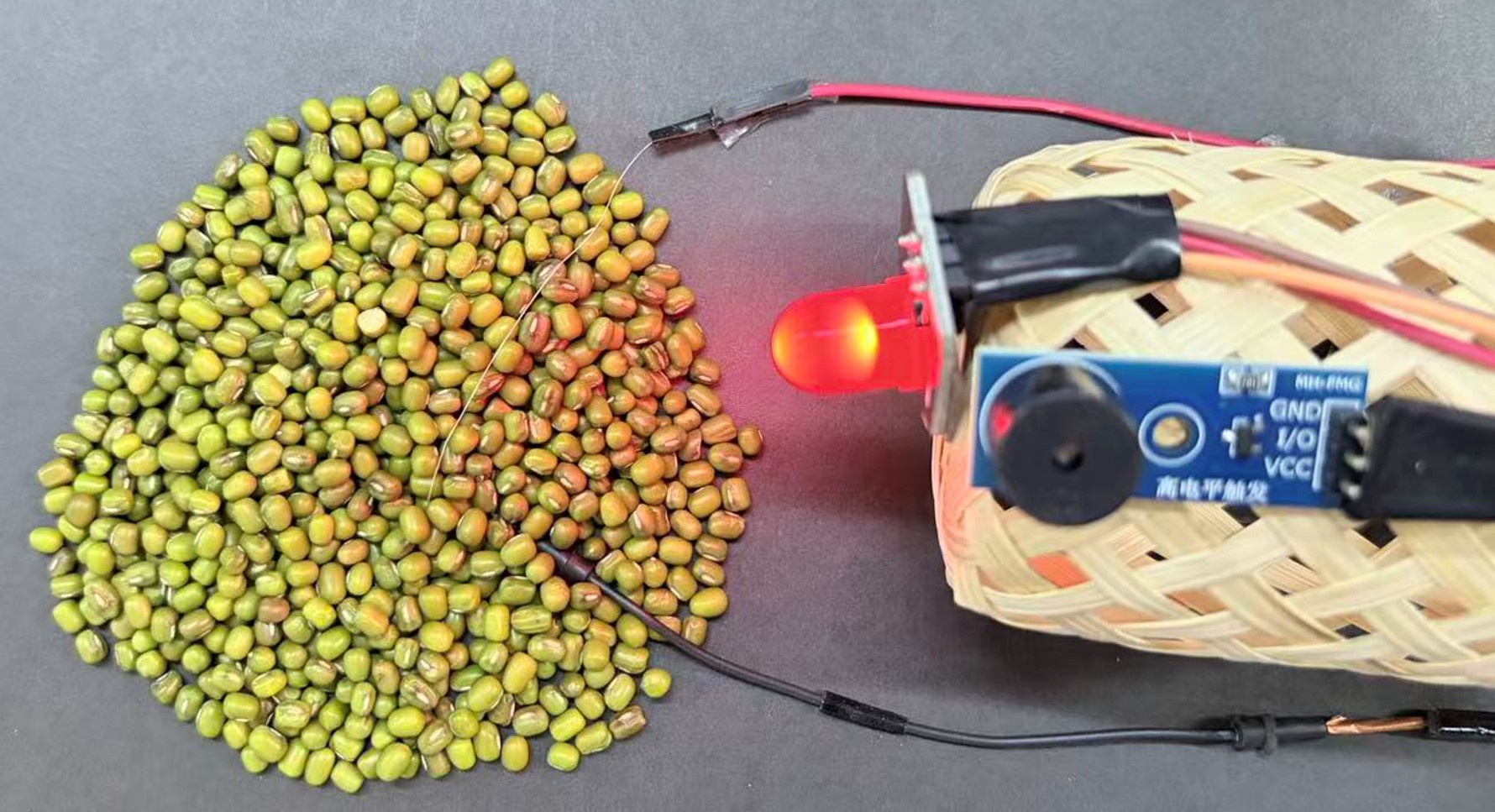
**

**Figure S23. Photograph of a linear type RAF sensor triggering an alarm at trace moisture detection point i.**

**
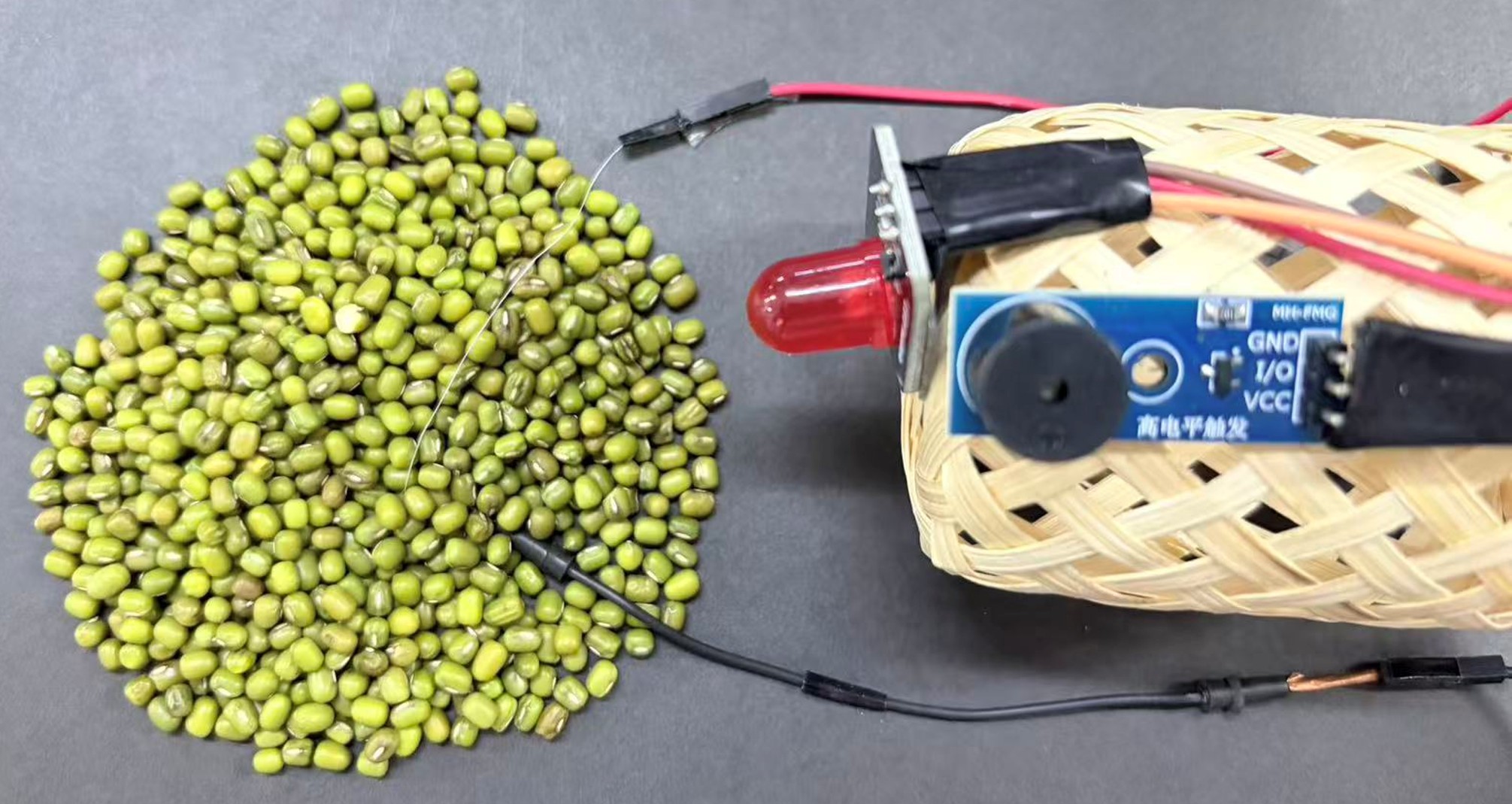
**

**Figure S24. Photograph showing the linear type RAF sensor failing to trigger an alarm at trace moisture detection point ii.**

**
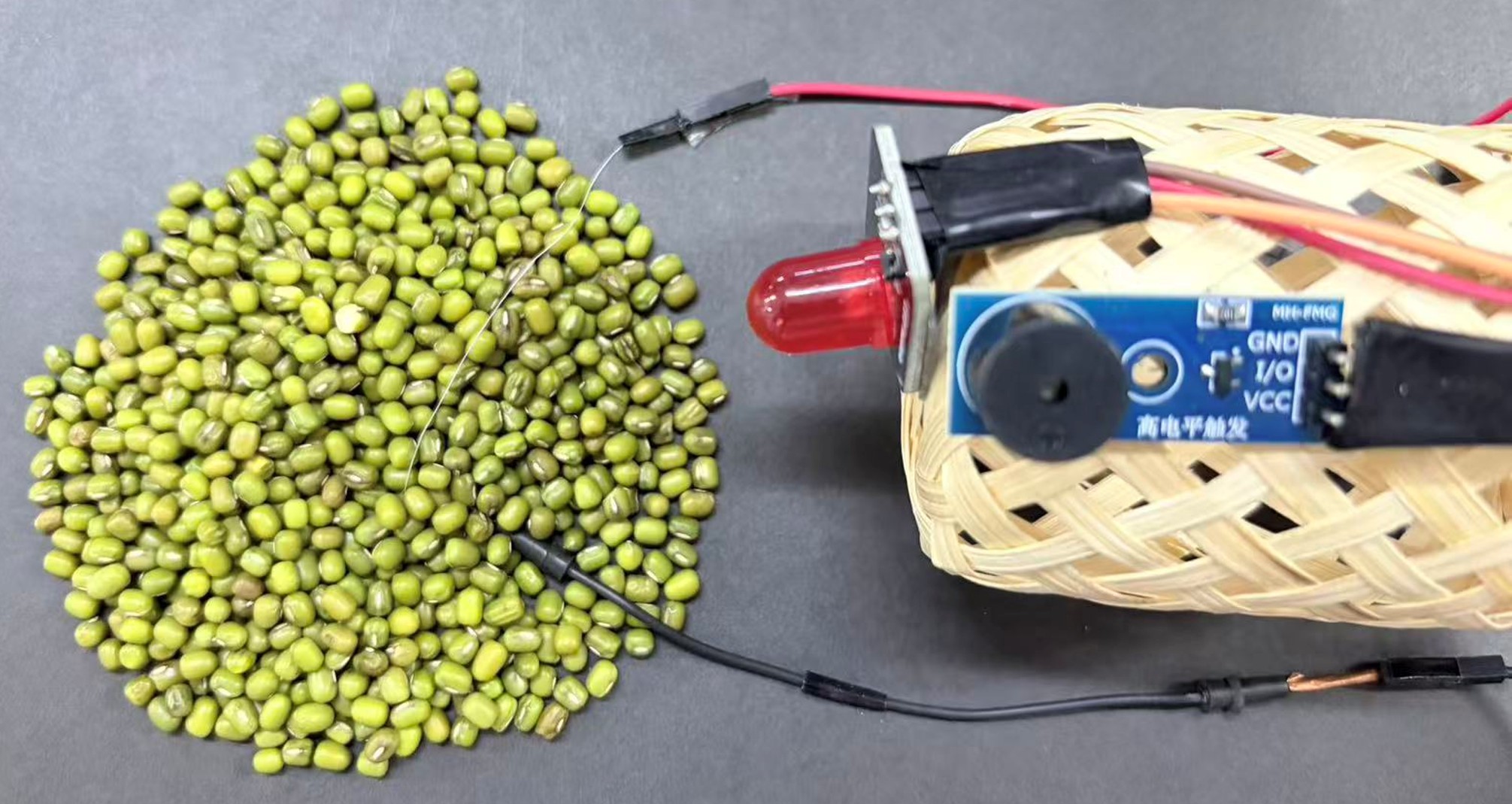
**

**Figure S25. Photograph showing the linear type RAF sensor failing to trigger an alarm at trace moisture detection point iii.**

**
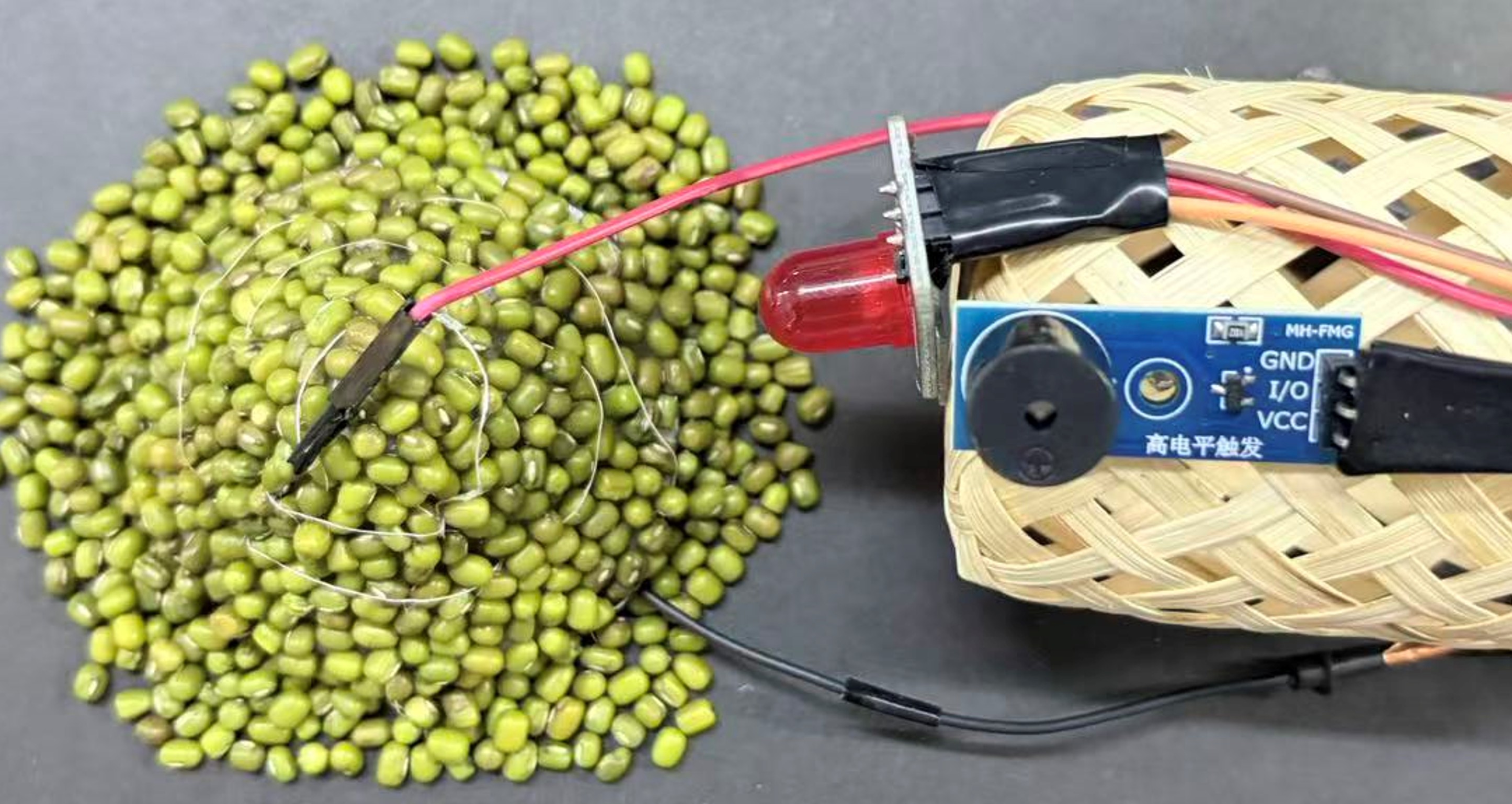
**

**Figure S26. Photograph showing the spiral type RAF sensor failing to trigger an alarm at the trace moisture detection point i.**

**
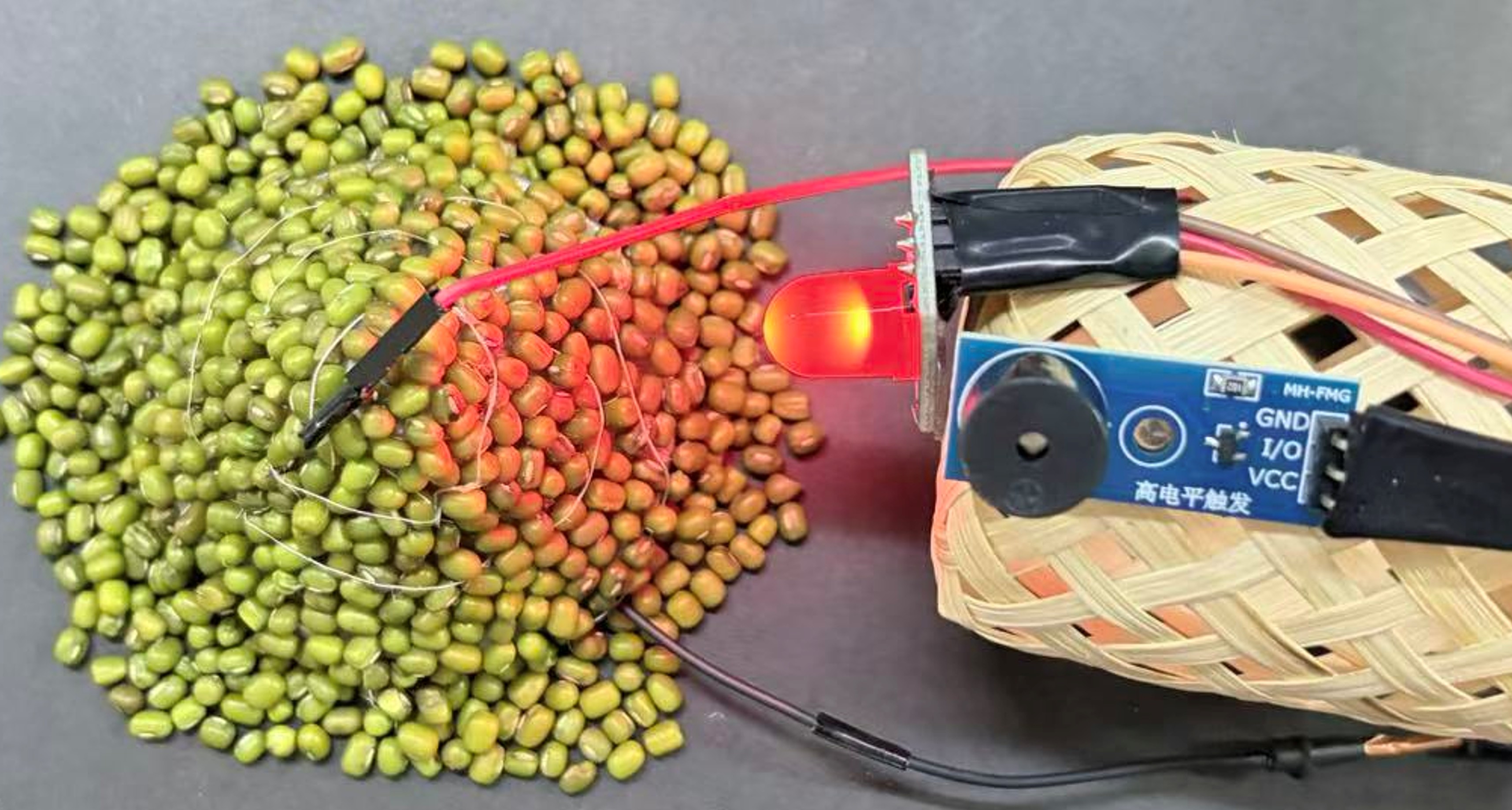
**

**Figure S27. Photograph of a spiral-type RAF sensor triggering an alarm at the trace moisture detection point ii.**

**
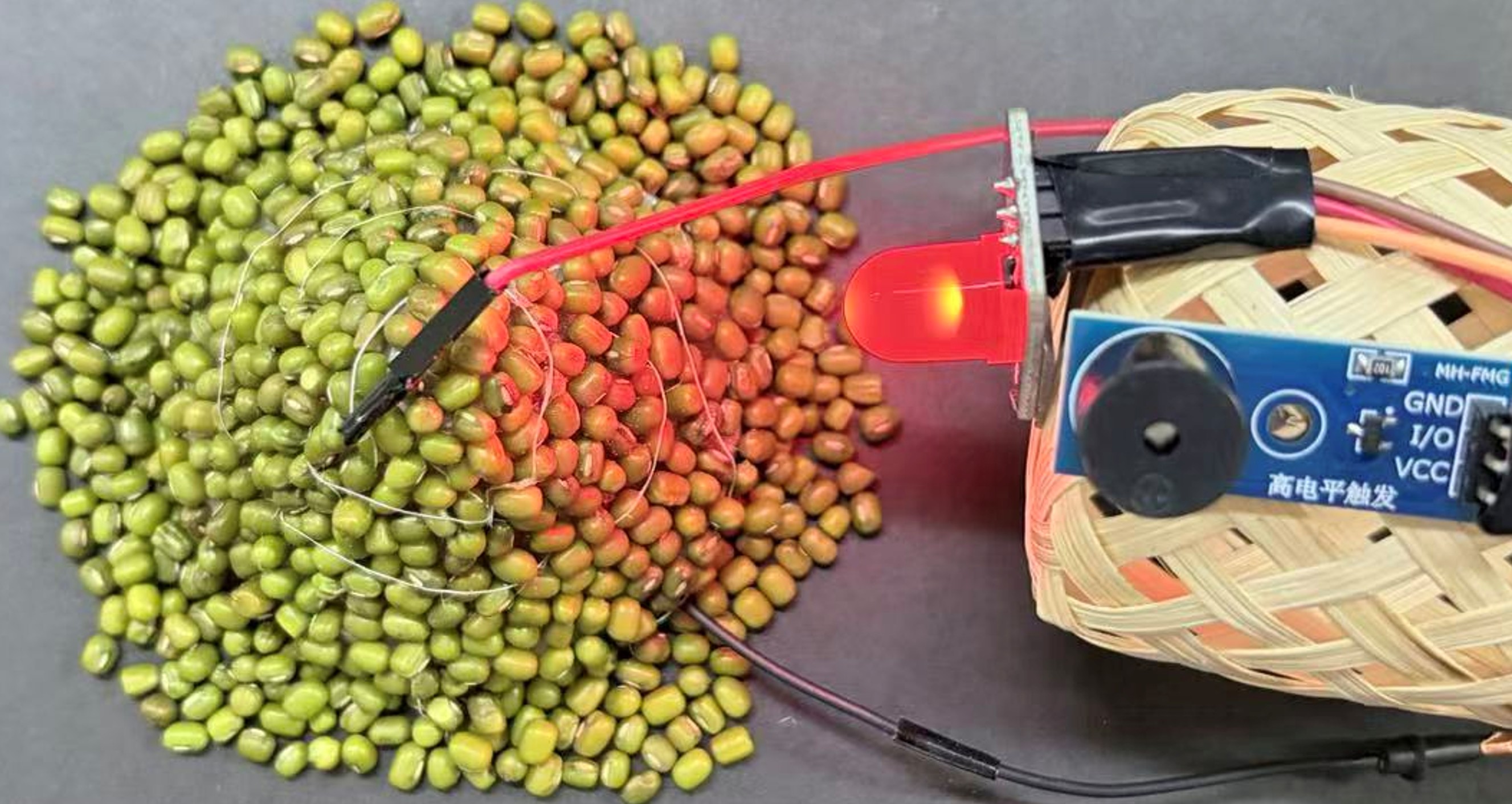
**

**Figure S28. Photograph of a spiral-type RAF sensor triggering an alarm at the trace moisture detection point iii.**

**
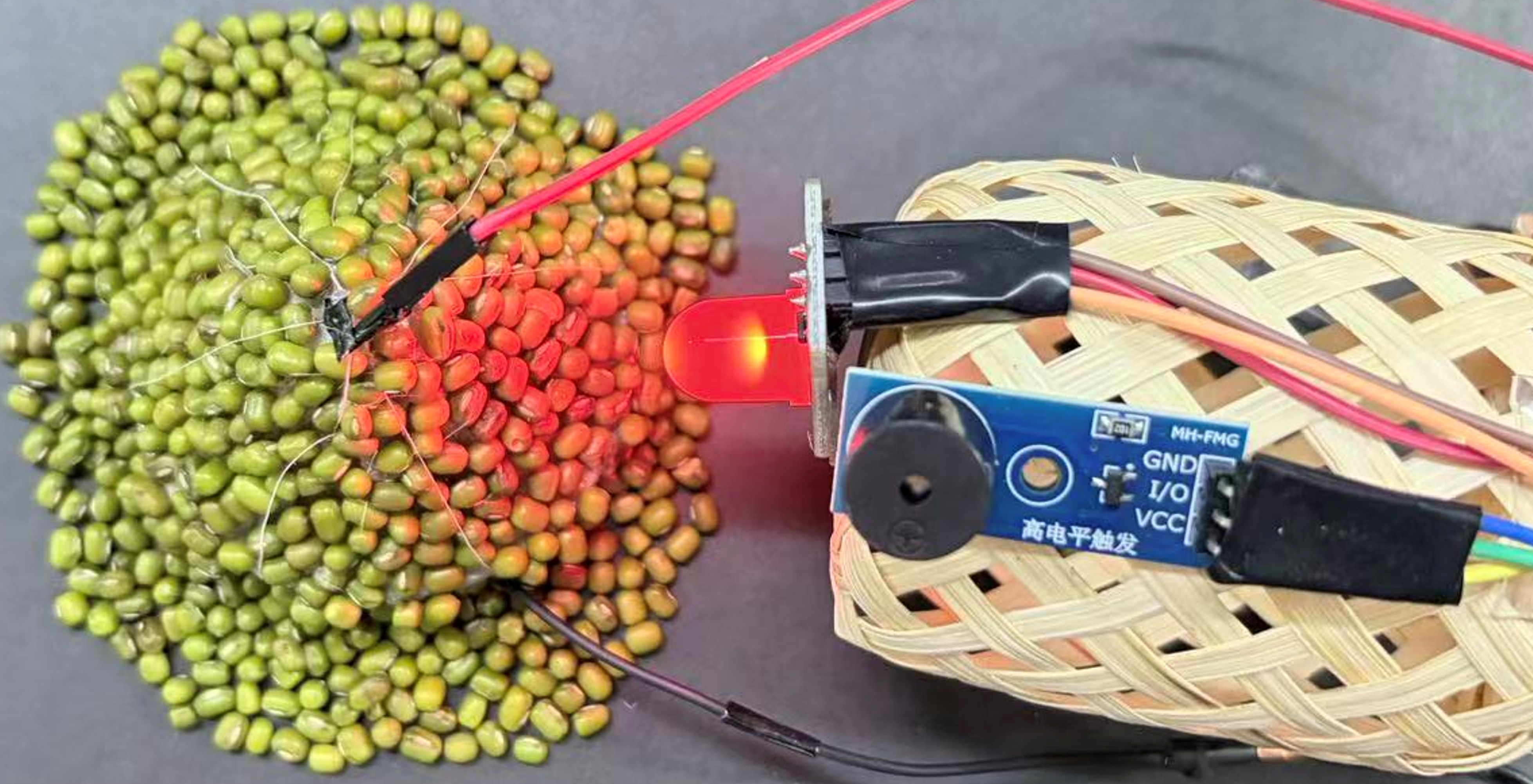
**

**Figure S29. Photograph of a branched type RAF sensor triggering an alarm at trace moisture detection point i.**

**
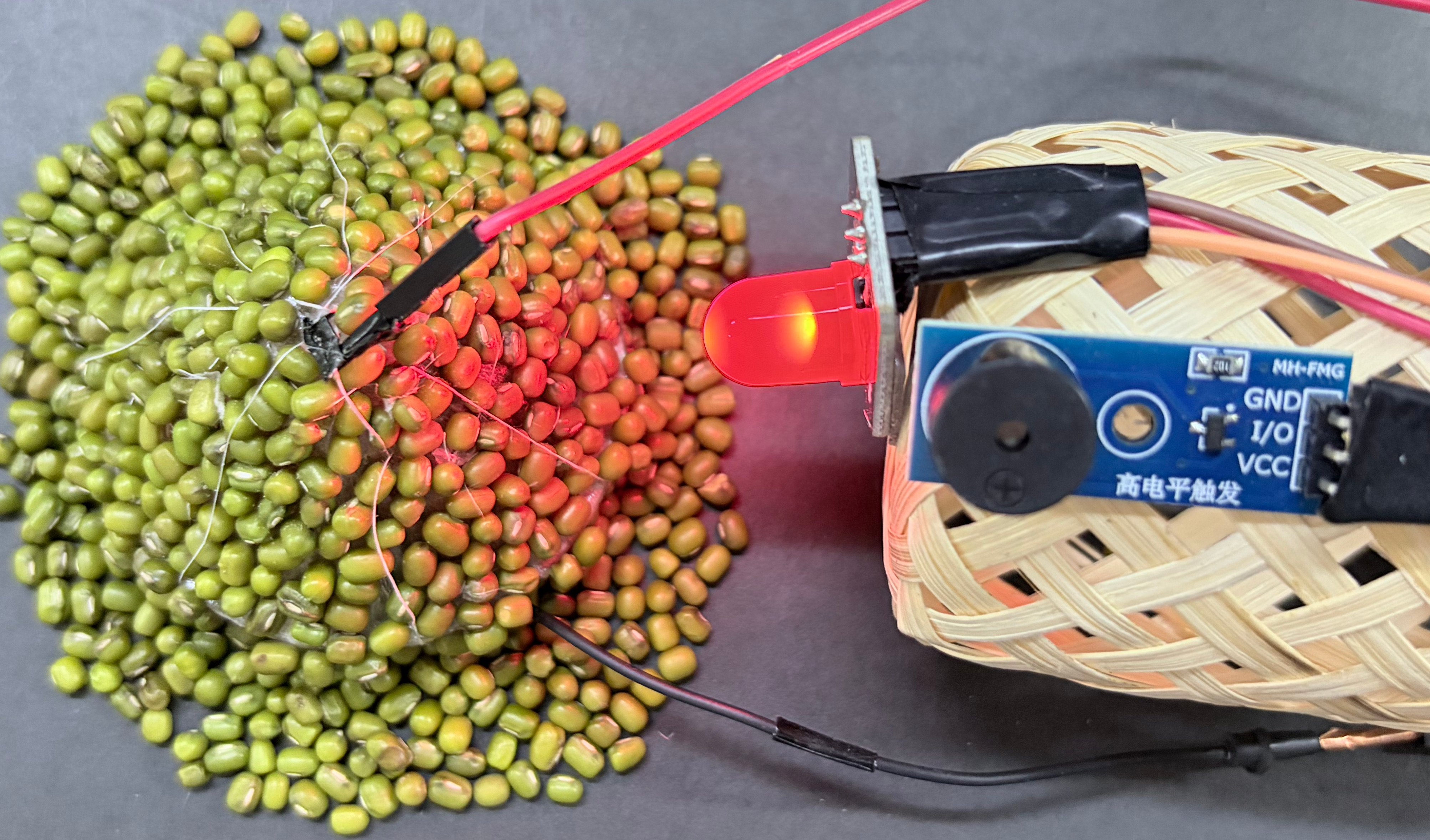
**

**Figure S30. Photograph of a branched type RAF sensor triggering an alarm at trace moisture detection point ii.**

**
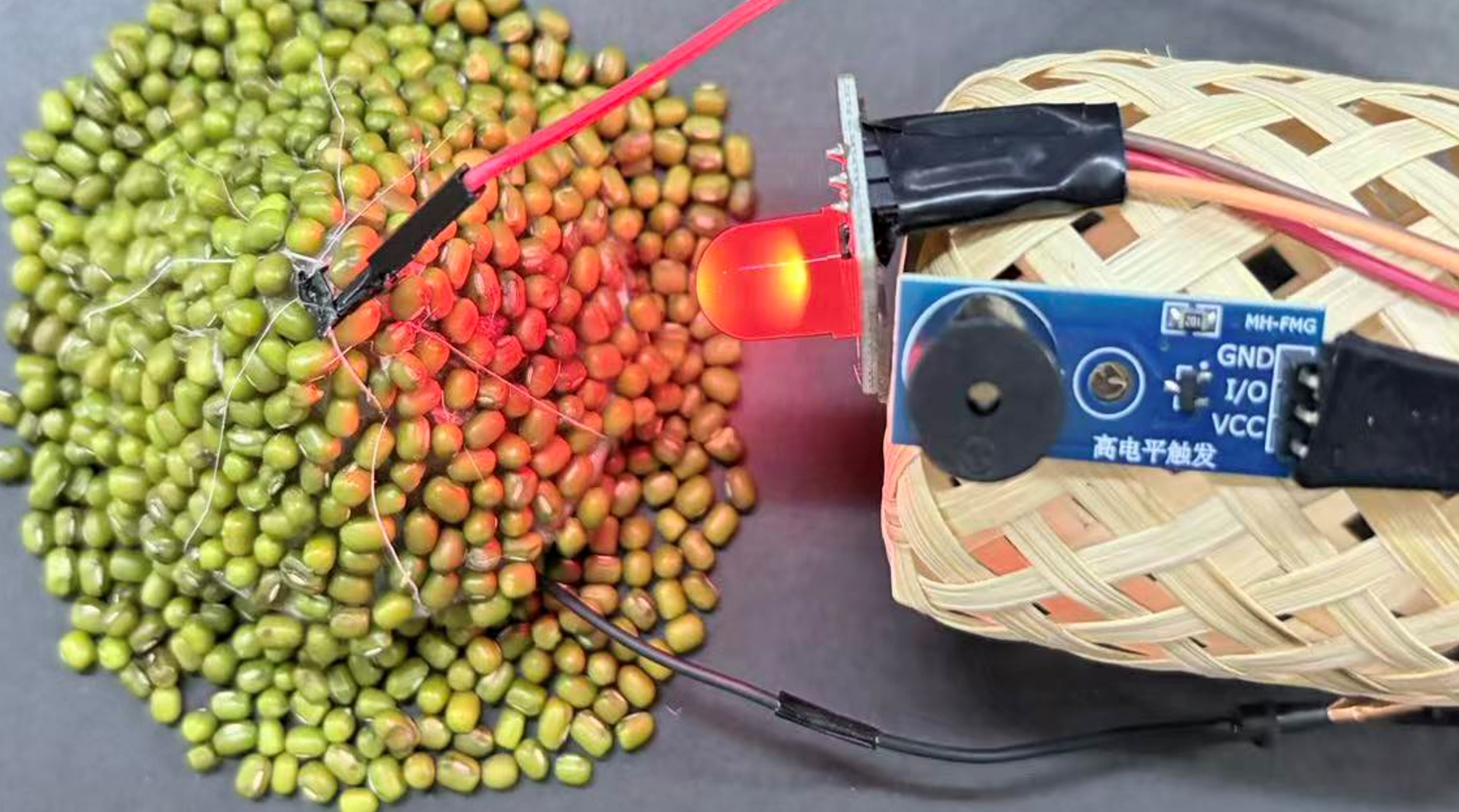
**

**Figure S31. Photograph of a branched type RAF sensor triggering an alarm at trace moisture detection point iii.**

**
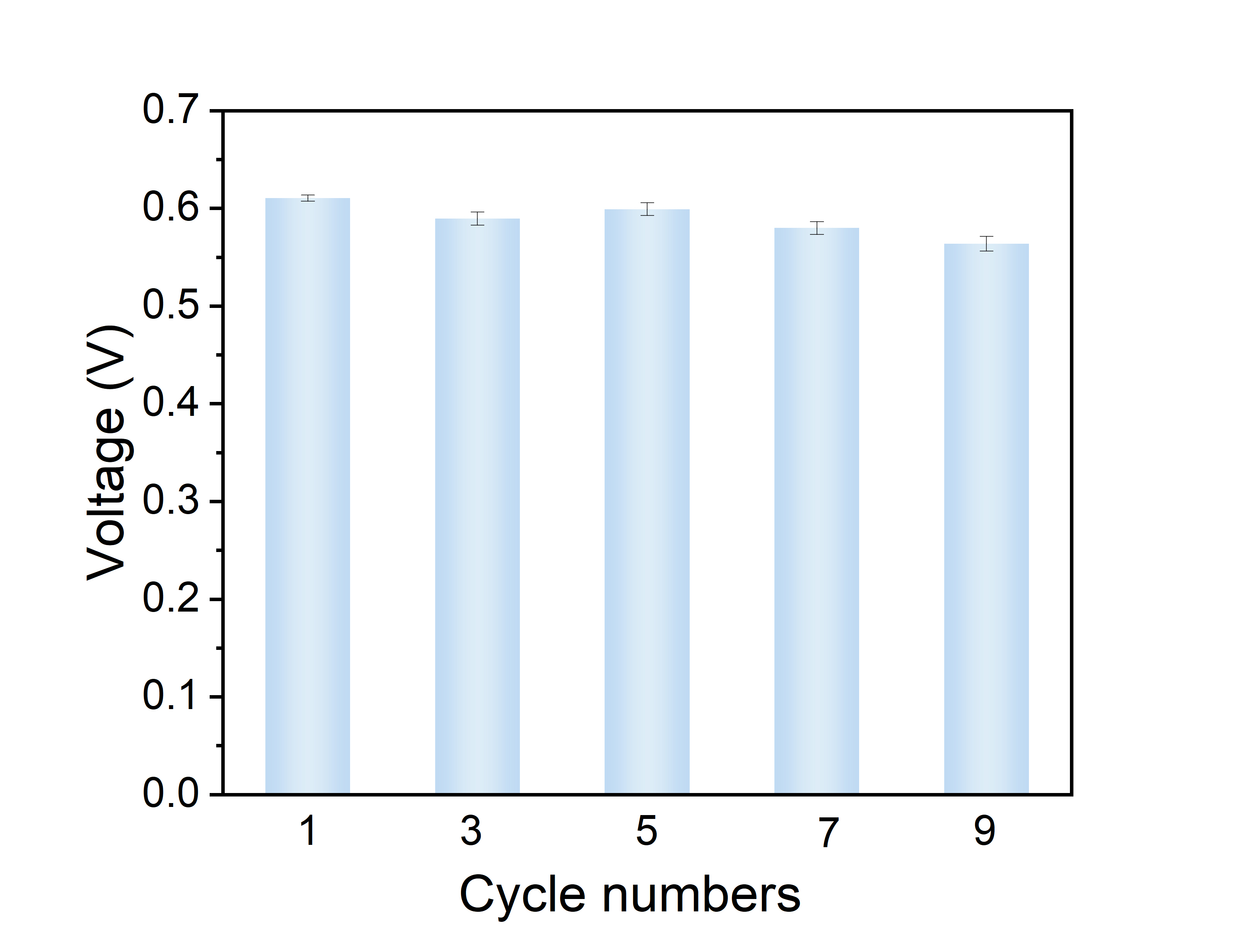
**

**Figure S32. Open‑circuit voltage response of RAF during cyclic testing.**

**
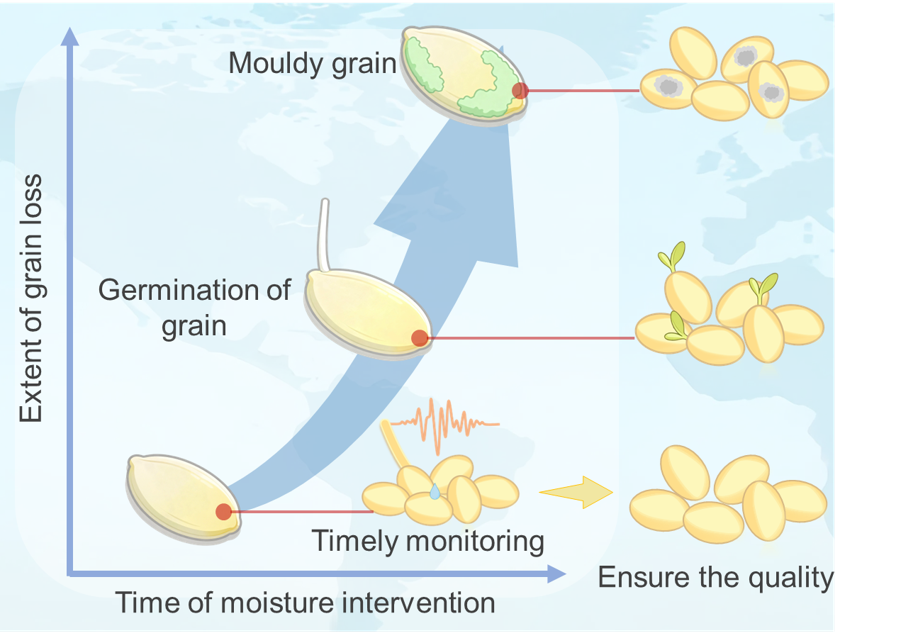
**

**Figure S33. Variation in grain loss over time under moisture intervention during grain transportation.**

**References**

[1] Ge C, Wang M, Zhou Y, Wang Y, Zhao F, Zhou C, Ma J, Wen F, Wang S, Liu M. Ion transport-triggered rapid flexible hydrovoltaic sensing. *Nat. Commun.* 2025; 16 (1): 8110.

[2] Zhan Y, Poisson J, Meng X, Wang Z, Chen L, Wu TH, Koehler R, Zhang K. Electrospun lignin/ZnO nanofibrous membranes for self–powered ultrasensitive flexible airflow sensor and wearable device. *Adv Mater.* 2025;37(37):2502211.

[3] Jiang H, Zhang H, Gao Z, Huang ZX, Qu Jp. Regenerative hydrovoltaic power generator with remarkable duration and fast power generation achieved by deep eutectic biomass complex. *Adv Mater*. 2026; 38(4): e17214.

[4] Deng M, Li Y, Yang X, Ren Z, Yin J, Zhang Q, Huang T, Yao Q, Zhou X, Wang L. A leaf‐inspired self‐powered humidity sensor with enhanced voltage output and rapid response/recovery. *Adv Funct Mater.* 2026: e26115.

[5] Zhang D, Shen D, Zhao G, Li F, Han Z, Han P, Huo J, Gu Q, Guo L, Tang R. An oryza sativa leaves‐inspired moisture electric generator for self‐powered wearable sensing and communication underwater. *Adv Funct Mater*. 2026: e74609.

[6] Zhong H, Wang S, Wang Z, Jiang J. Asymmetric self-powered cellulose-based aerogel for moisture-electricity generation and humidity sensing. *Chem Eng J*. 2024; 486: 150203.

[7] Brunauer S, Emmett P H, Teller E. Adsorption of gases in multimolecular layers. J. Am. Chem. Soc. 1938; 60 (2): 309-319.
